# New Organic Compounds Against *Scedosporium* and Mucorales: Antifungal Activity and Mechanisms of Cell Wall and Membrane Disruption

**DOI:** 10.3390/molecules31203691

**Published:** 2026-10-15

**Authors:** Rodrigo Rollin-Pinheiro, Yuri de Castro-Almeida, Mariana Ingrid Dutra da Silva Xisto, Johnatha de Souza-Santos, Daniel Clemente de Moraes, Bruno Vicente, Victor Midlej, Fabiano Travanca Toledo, Renan Ferrarini, Glaucio Monteiro Ferreira, Alcindo A. Dos Santos, Eliana Barreto-Bergter, Antonio Ferreira-Pereira

**Affiliations:** 1 Departamento de Microbiologia, Imunologia e Parasitologia, Faculdade de Ciências Médicas, Universidade do Estado do Rio de Janeiro (UERJ), Rio de Janeiro 20550-170, Brazil; 2 Departamento de Microbiologia Geral, Instituto de Microbiologia Paulo de Góes, Universidade Federal do Rio de Janeiro (UFRJ), Rio de Janeiro 21941-902, Brazil; 3 Laboratório de Biologia Estrutural, Instituto Oswaldo Cruz, Fundação Oswaldo Cruz (Fiocruz), Rio de Janeiro 21040-900, Brazil; 4 Department of Fundamental Chemistry, Institute of Chemistry, University of São Paulo, São Paulo 05508-000, Brazil

**Keywords:** antifungal agents, organochalcogenides, *Scedosporium*, *Lomentospora prolificans*, Mucorales, drug-resistant fungi, membrane permeability, antifungal drug discovery

## Abstract

The emergence of infections caused by *Scedosporium/Lomentospora* and Mucorales species poses a major clinical challenge due to their intrinsic resistance to multiple antifungal drugs and the limited number of therapeutic options available. In this study, we evaluated the antifungal activity of synthetic organic compounds and characterized the mechanisms of antifungal activity. Eight compounds were initially screened against *Scedosporium aurantiacum* and *Rhizopus oryzae*. Compounds **2**, **3**, and **4** inhibited more than 60% of fungal growth and were selected for further characterization. Antifungal susceptibility assays revealed that compound **2** exhibited the strongest activity, with minimum inhibitory concentrations ranging from 12.5 to 25 μg/mL, whereas compounds **3** and **4** showed MIC values between 50 and 100 μg/mL. All compounds displayed fungistatic activity and significantly impaired fungal germination and biofilm development. Drug interaction analyses demonstrated additive or synergistic effects when the compounds were combined with clinically used antifungals. Investigations revealed that the compounds induced alterations in the fungal cell surface, including reductions in neutral lipids and chitin, increased membrane permeability, disruption of sterol-enriched membrane microdomains, and ultrastructural abnormalities. Exogenous ergosterol partially reversed the activity of compounds **2** and **3**, suggesting interactions with membrane sterols. In silico toxicity prediction indicated generally favorable safety profiles. In summary, these findings identify synthetic organic compounds as promising scaffolds for antifungal drug discovery and highlight their potential to target fungal membrane and cell wall integrity in resistant filamentous pathogens.

## 1. Introduction

Fungal infections have been gradually emerging worldwide over the last decades, due to the increasing number of individuals with underlying diseases, such as diabetes, cancer, HIV/AIDS and other hematological and immunosuppressive conditions [1]. Currently, 6.5 million cases of invasive fungal infections and 3.8 million deaths are estimated annually [2]. Among fungal pathogens, two groups of filamentous fungi are particularly relevant due to their difficult-to-treat nature: *Scedosporium* and *Lomentospora* species and the order Mucorales.

*Scedosporium/Lomentospora* complex is composed of septate filamentous fungi widely distributed in nature, especially associated with human-impacted areas due to their adaptability to environments with high osmotic pressure and high nitrogen concentrations [3]. In general, five species are more frequently associated with human infections: *Scedosporium aurantiacum*, *Scedosporium boydii*, *Scedosporium apiospermum*, *Scedosporium dehoogii* and *Lomentospora prolificans*. Scedosporiosis and lomentosporiosis have a variety of clinical manifestations and can be acquired through the traumatic inoculation of conidia and mycelial fragments, which can affect both healthy and immunocompromised patients and lead to subcutaneous infections, or through the inhalation of conidia, which causes infection mainly in immunocompromised individuals who may develop life-threatening invasive disease [3].

The Mucorales group is composed of non-septated filamentous fungi found in decomposing organic matter or soil [4]. The most frequent Mucorales genera associated with human infections are *Rhizopus*, *Lichtheimia* and *Mucor* [5]. Mucormycosis is an invasive fungal infection associated with high mortality rates due to its aggressive nature and difficulties in diagnosis [6]. Several risk factors are associated with the development of mucormycosis, such as diabetes, chemotherapy, organ transplantation, hematological diseases and high serum iron levels [7]. The aggressive characteristics of mucormycosis are related to its thermotolerance, rapid growth, ability to bind to endothelial cells and ability to acquire iron from the host [6]. The most common clinical manifestations are rhinocerebral, pulmonary, cutaneous, gastrointestinal, and disseminated infections [4]. Angioinvasion is an important characteristic of invasive mucormycosis [7]. Penetration of the endothelium leads to thrombosis and tissue necrosis, facilitating hematogenous dissemination of the fungi [7].

A concerning factor contributing to the poor prognosis of scedosporiosis, lomentosporiosis and mucormycosis is their resistance to several antifungal drugs available in clinical practice. Mucorales are resistant to most antifungal drugs, such as amphotericin B, posaconazole and isavuconazole [8,9], although these drugs are still used to treat mucormycosis. *Scedosporium* and *Lomentospora* species display resistance to amphotericin B, itraconazole, fluconazole and echinocandins, and the most recommended drug for treating scedosporiosis and lomentosporiosis is voriconazole [3,10]. Thus, the treatment of fungal infections is limited not only by pathogen resistance but also by the low diversity of available antifungal agents and their significant toxicity.

**Figure 1:**
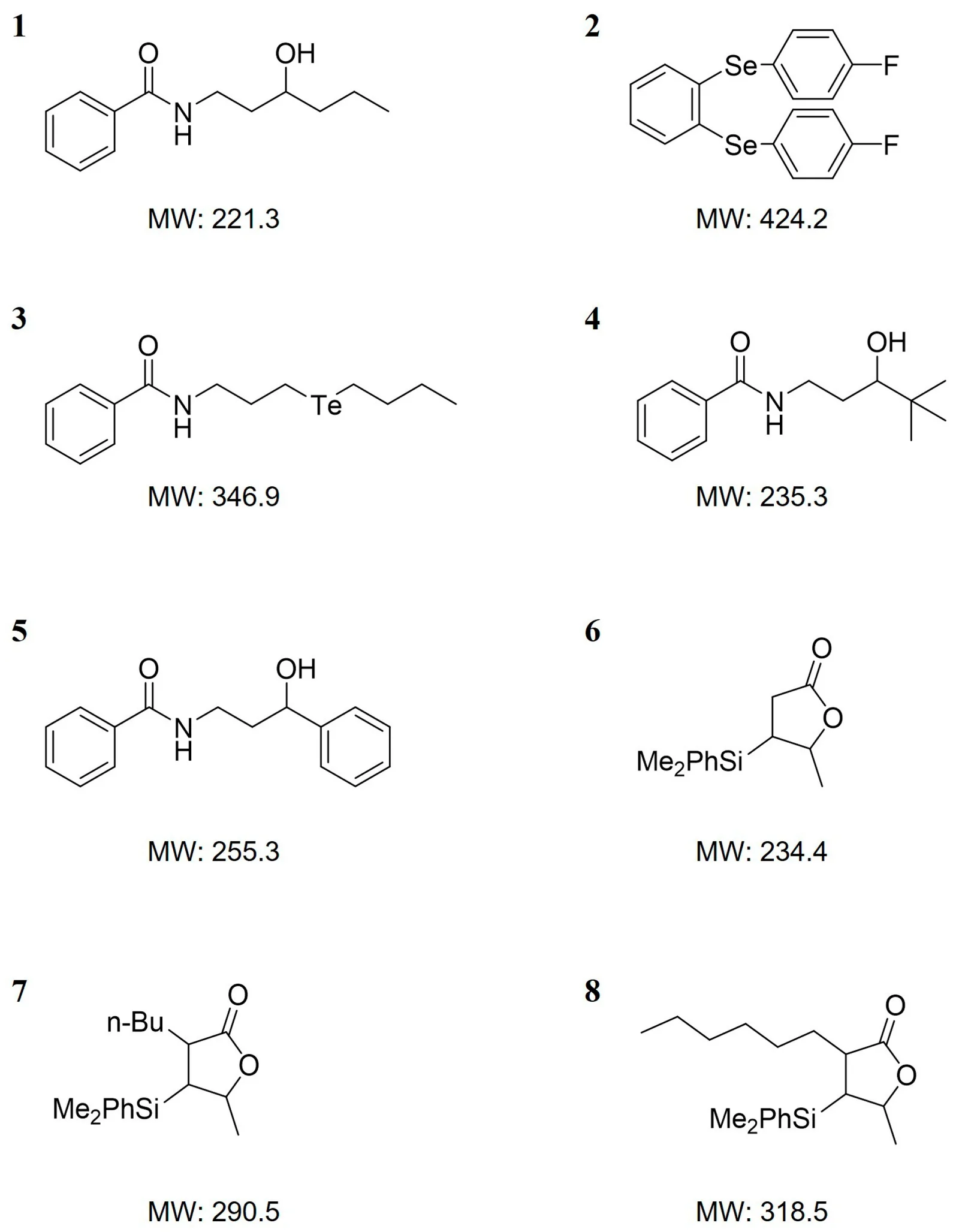
Chemical structures of synthetic organic compounds used in the study. MW: molecular weight, expressed in g/mol.

In this context, the study of new synthetic compounds is useful for investigating novel molecules that may target different structures in fungal cells. In previous studies, some of these compounds, derived from a chemical library developed by our group, have already been described as inhibitors of efflux pumps in yeasts, such as *Candida albicans* and *Saccharomyces cerevisiae*, but their effects on the growth of filamentous fungal pathogens have not been reported to date [11,12]. Considering the importance of evaluating the broad spectrum of activity of antifungal compounds and the clinical relevance of filamentous fungi, the present study aimed to investigate the antifungal properties of some compounds from the same library (Figure 1) against two representative groups of emerging filamentous fungi: Scedosporium and Mucorales species.

## 2. Results

### 2.1. Screening and Identification of Synthetic Compounds

A total of eight compounds were screened using *S. aurantiacum* and *R. oryzae* as representative species. Their chemical structures are shown in Figure 1. Compounds **2**, **3** and **4** inhibited more than 50% of *S. aurantiacum* growth, whereas seven compounds (**2** to **8**) inhibited at least 50% of *R. oryzae* growth, with the exception of compound **1** (Table 1). Since compounds **2**, **3** and **4** were active against both fungal pathogens tested, they were selected to continue with the study.

### 2.2. Minimum Inhibitory Concentration of Selected Compounds Against Different Scedosporium and Mucorales Species

To investigate the minimum inhibitory (MIC) and fungicidal (MFC) concentrations of compounds **2**, **3** and **4**, an antifungal susceptibility test was performed using serial dilutions of each compound.

Compound **2** was found to be active at concentrations between 12.5 and 25 μg/mL against almost all fungal species tested, except for *M. velutinosus*, against which it was not active, and *Cunninghamella* sp., for which it presented an MIC value of 100 μg/mL (Table 2). Compounds **3** and **4** displayed MIC values between 50 and 100 μg/mL for almost all fungal species, except for *M. velutinosus*, which was resistant to all concentrations used (Table 2). Regarding the MFC analyses, none of the three compounds showed fungicidal activity against any of the fungal species tested, suggesting that they display fungistatic activity (Table 2).

### 2.3. Influence of Synthetic Compounds on Fungal Germination

**Figure 2:**
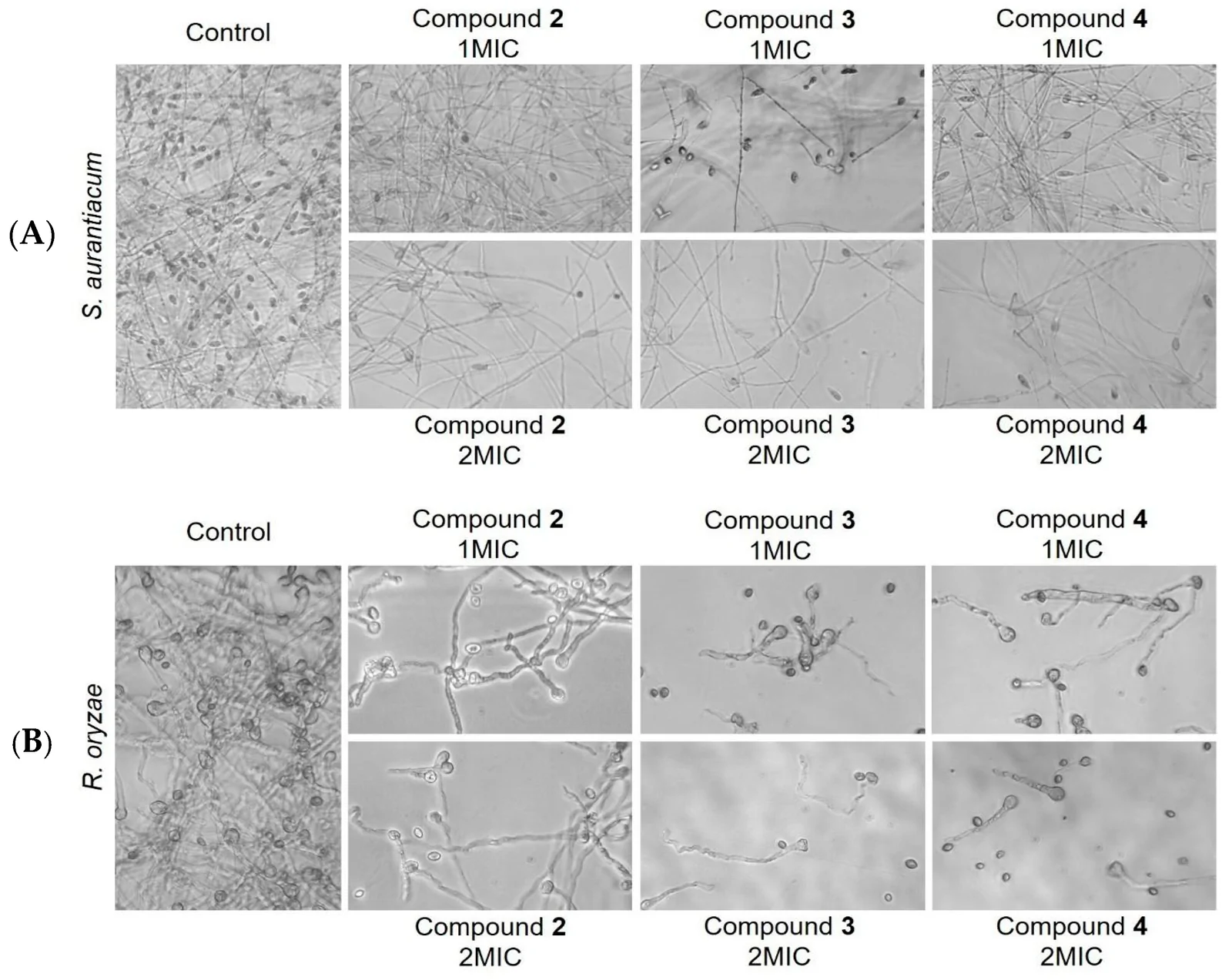
Light microscopy of *S. aurantiacum* (**A**) and *R. oryzae* (**B**) germination after treatment with compounds **2**, **3** and **4**. Control represents biofilm growth in the absence of any compound.

Since compounds **2**, **3** and **4** displayed antifungal activity against different species, their effects on fungal germination were evaluated. *S. aurantiacum* and *R. oryzae* were used as representative species. Whereas the control grown in the absence of any compound presented a dense mycelial growth in the culture medium, the treatment of *S. aurantiacum* with all three compounds resulted in a reduction in fungal growth, mainly at 2 MIC, which suggests that the germination process was impaired. In *R. oryzae*, a decrease in fungal growth was observed when 1 MIC and 2 MIC concentrations of all three compounds were used (Figure 2).

### 2.4. Effect of Synthetic Compounds Against Fungal Biofilm

**Figure 3:**
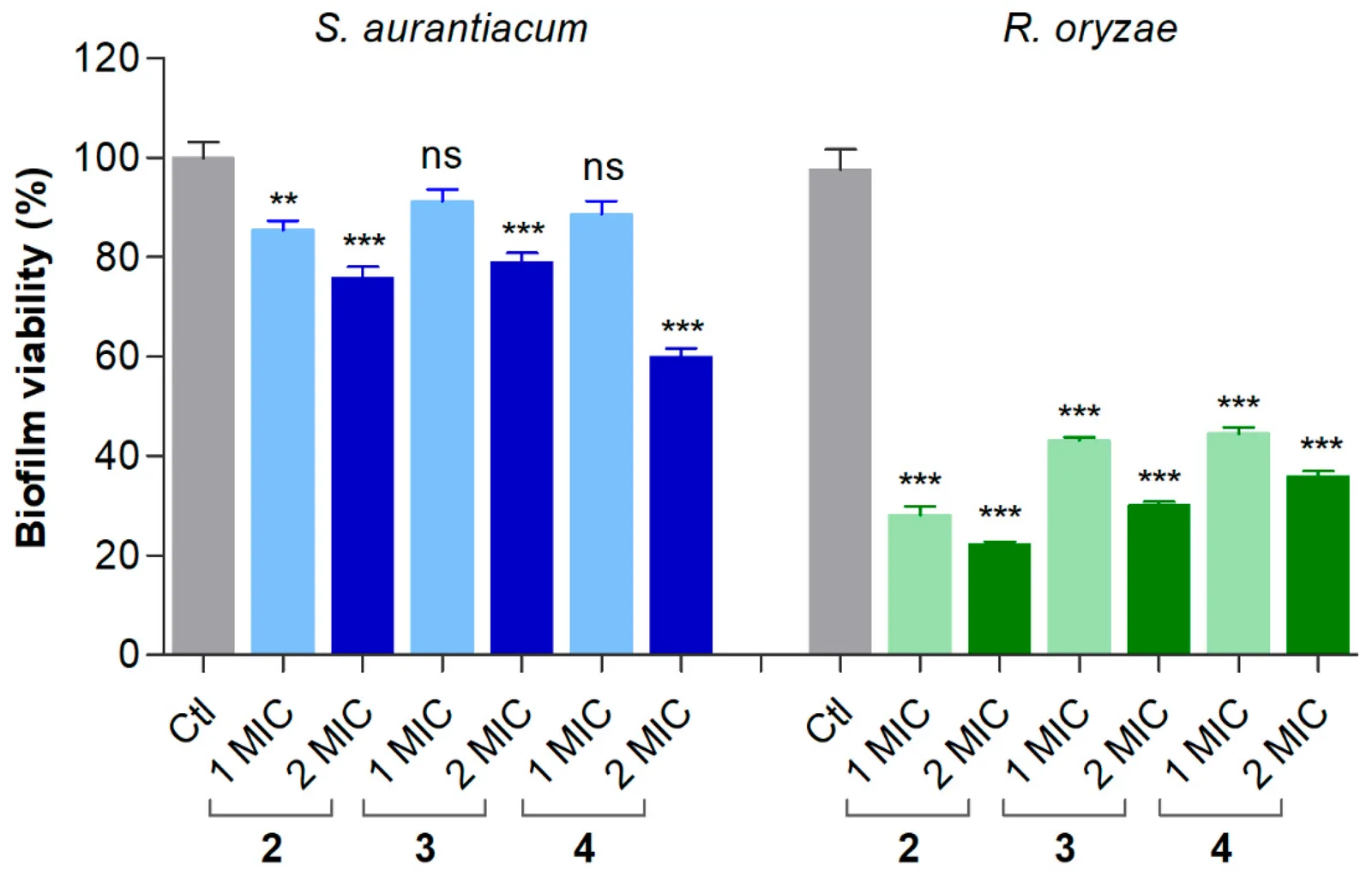
*S. aurantiacum* and *R. oryzae* biofilm viability after treatment with 1 MIC or 2 MIC of compounds **2**, **3** and **4**. Control (Ctl): fungal growth in the absence of any compound. ns: not significant (*p* > 0.05), ** *p* < 0.001, *** *p* < 0.0001.

To evaluate the effect of compounds **2**, **3** and **4** on fungal biofilms, *S. aurantiacum* and *R. oryzae* were used as representative species. In *S. aurantiacum*, compound **2** significantly reduced biofilm viability at 1 MIC and 2 MIC, whereas compounds **3** and **4** decreased fungal biofilm viability only at 2 MIC. In *R. oryzae*, all three compounds reduced more than 50% of biofilm viability at both concentrations used (Figure 3).

### 2.5. Interaction of Synthetic Compounds with Antifungal Drugs Used in Clinical Settings

To investigate whether the synthetic compounds could positively or negatively interact with antifungal drugs used in clinical settings for the treatment of *Scedosporium* and Mucorales infections, analyses based on the Fractional Inhibitory Concentration Index (FICI) and Bliss Independence methods were performed. *S. aurantiacum* and *R. oryzae* were used as representative species. Voriconazole and caspofungin were used to evaluate their interactions in *S. aurantiacum*, whereas posaconazole and amphotericin B were used with *R. oryzae*.

Using the FICI method, the combinations of voriconazole/compound **3** and caspofungin/compound **2** were additive in *S. aurantiacum*, whereas all other combinations were indifferent in this fungus (Table 3). For *R. oryzae*, a synergistic effect was observed between amphotericin B and compound **2**, but all the other combinations showed an indifferent effect (Table 3).

When the BLISS method was performed, all combinations of voriconazole or caspofungin with compounds **2**, **3** or **4** were synergistic for *S. aurantiacum*. Regarding *R. oryzae*, similar results were observed, except for the combination of amphotericin B and compound **4**, which displayed an antagonistic effect (Table 4).

### 2.6. Toxicity Prediction

According to the Osiris Property Explorer, the compounds exhibit distinct toxicity profiles. Compound **2** showed no predicted toxicity risks. On the other hand, compound **3** was predicted as tumorigenic, while compound **4** presented a reproductive effect alert (Table 5). Risks of mutagenicity and irritative effects were not detected for any of the tested compounds. Considering the control antifungals, voriconazole and amphotericin B were not predicted as mutagenic, tumorigenic, irritant, or having a reproductive effect. Posaconazole was associated with mutagenicity and tumorigenicity.

Data obtained using GUSAR are presented in Table 6. LD_50_ values for compounds **2**, **3**, and **4** ranged from 93.6 to 2059.0 mg/kg, depending on the route of administration. Compound **4** was predicted to be the safest compound. The three tested compounds exhibited higher LD_50_ values than the control antifungals for oral and subcutaneous administration. Considering intravenous administration, the compounds displayed higher LD_50_ values than posaconazole and amphotericin B.

### 2.7. Cellular Alterations Using Fluorescent Probes

To investigate some cellular alterations, mainly on the cell surface, caused by compounds **2**, **3** and **4**, *S. aurantiacum* and *R. oryzae* were used as representative species, and Nile red and calcofluor were used to evaluate the content of neutral lipids and chitin, respectively.

**Figure 4:**
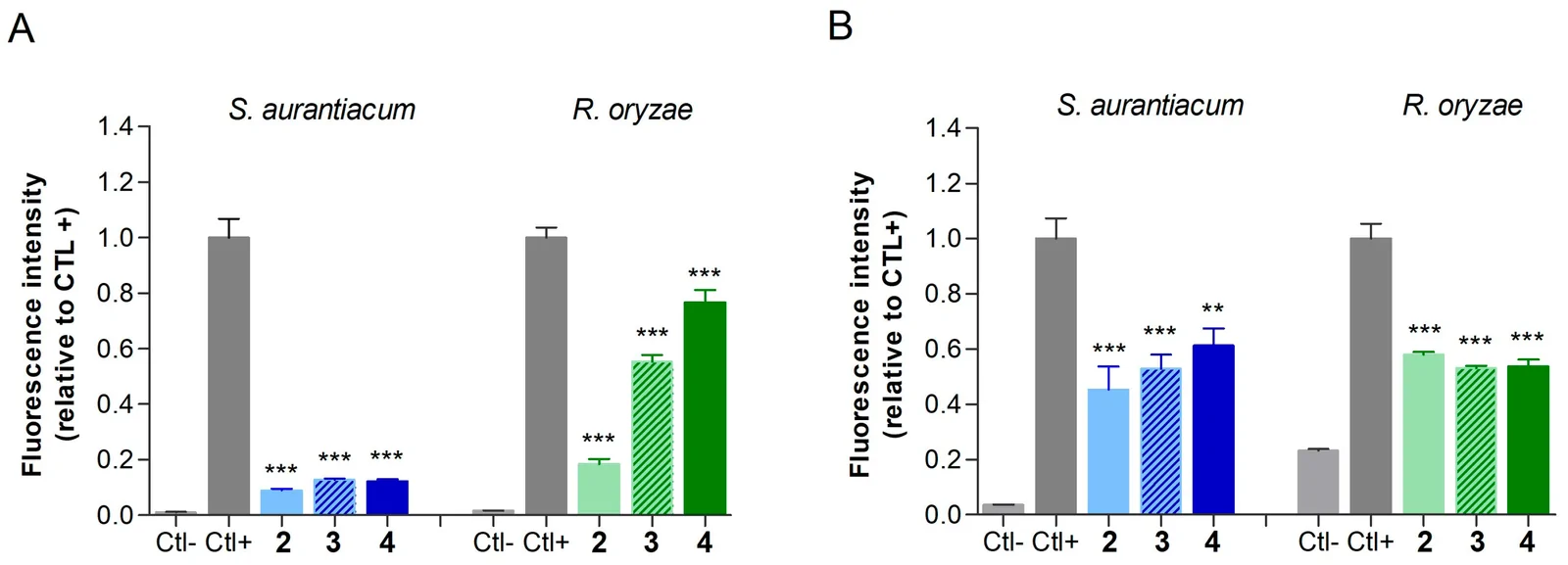
Nile red (**A**) and calcofluor (**B**) staining of *S. aurantiacum* and *R. oryzae* cells when treated with compounds **2**, **3** and **4**. Ctl(−): negative control with no fluorescent probe. Ctl(+): positive control of fluorescence with no treatment with compounds. **2**: compound **2**; **3**: compound **3**; **4**: compound **4**. ** *p* < 0.001, *** *p* < 0.0001.

Compared to the control, fungal neutral lipids were reduced when cells were treated with all three compounds. In *S. aurantiacum*, Nile red staining decreased to less than 20%, whereas in *R. oryzae* it reduced to 20, 60 and 80% when treated with compounds **2**, **3** and **4**, respectively (Figure 4A).

Regarding calcofluor staining, the treatment with all three compounds led to a reduction in chitin content of around 40–60% in both *S. aurantiacum* and *R. oryzae* (Figure 4B).

### 2.8. Influence of Synthetic Compounds on Fungal Susceptibility to Cell Stressors

To investigate whether the compounds influence the susceptibility of *S. aurantiacum* and *R. oryzae* to some cell stressors, fungal cells treated with compounds **2**, **3** or **4** were grown in the presence of sodium dodecyl sulfate (SDS), NaCl and menadione, known as membrane, cell wall and oxidative stressors, respectively.

**Figure 5:**
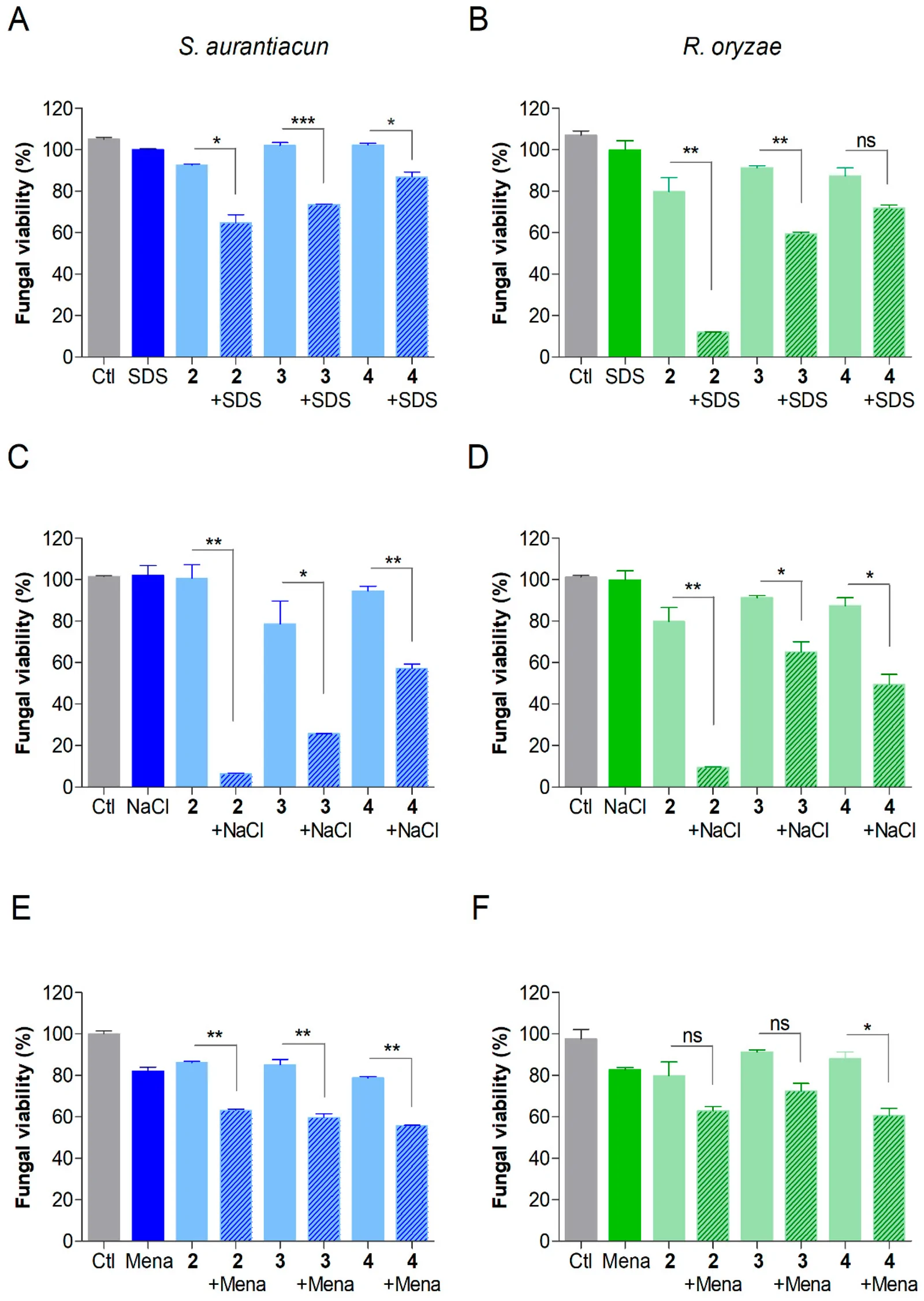
Susceptibility of *S. aurantiacum* (**A**,**C**,**E**) and *R. oryzae* (**B**,**D**,**F**) to SDS (**A**,**B**), NaCl (**C**,**D**) and menadione (**E**,**F**) when treated with compounds **2**, **3** or **4**. Control (Ctl): fungal cells in the absence of cell stressors and any compounds. Bars of SDS, NaCl and menadione alone represent fungal cells grown in the presence of sub-inhibitory concentrations of cell stressors, but in the absence of any compounds. Mena: menadione. **2**: compound **2**; **3**: compound **3**; **4**: compound **4**. ns: not significant (*p* > 0.05), * *p* < 0.01, ** *p* < 0.001, *** *p* < 0.0001.

In *S. aurantiacum*, all three compounds caused a reduction of 20−40% in fungal viability in the presence of SDS, of 50−90% with NaCl and around 40% when menadione was added (Figure 5A,C,E). In *R. oryzae*, compounds **2** and **3** enhanced fungal susceptibility to SDS and NaCl, but not to menadione, whereas compound **4** increased fungal susceptibility to NaCl and menadione, but not to SDS (Figure 5B,D,F).

### 2.9. Influence of Synthetic Compounds on Fungal Membrane Permeability

Considering that all three compounds caused alterations in fungal surface parameters, such as neutral lipids and chitin contents, and influenced susceptibility to surface stressors, such as SDS and NaCl, the membrane permeability of *S. aurantiacum* and *R. oryzae* was evaluated through the measurement of extracellular DNA in the growth medium and the use of Sytox Green.

**Figure 6:**
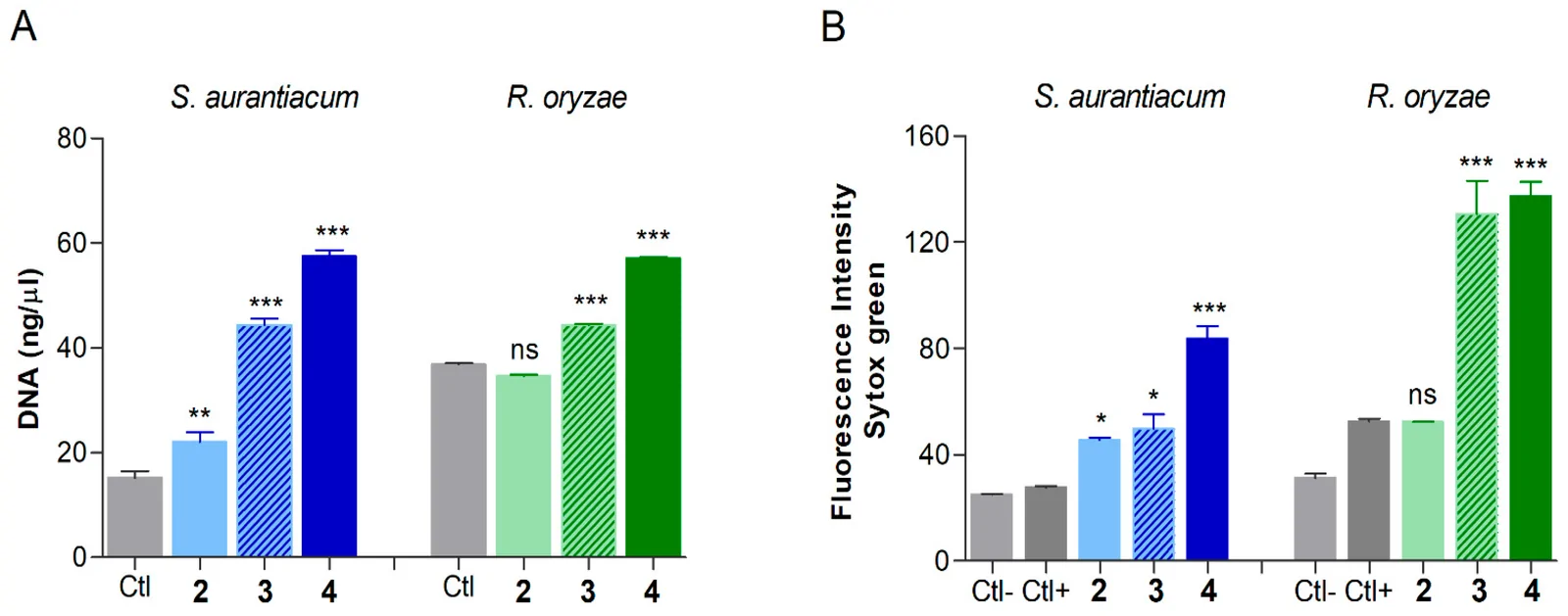
Fungal cell permeability after treatment with compounds **2**, **3** and **4**, evaluated by extracellular DNA measurement (**A**) and Sytox Green staining (**B**). Control (Ctl): fungal cells in the absence of any compounds. Ctl(−): cell with no Sytox Green staining. Ctl(+): cells stained with Sytox Green, but grown in the absence of any compounds. **2**: compound **2**; **3**: compound **3**; **4**: compound **4**. ns: not significant (*p* > 0.05), * *p* < 0.01, ** *p* < 0.001, *** *p* < 0.0001.

All three compounds increased extracellular DNA in both fungi, reaching around 3-fold higher DNA levels in *S. aurantiacum* treated with compound **4** and 2-fold higher DNA levels in *R. oryzae* treated with the same compound **4**, compared to the control (Figure 6A). Regarding the use of Sytox Green, which stains fungal nuclei when the plasma membrane is more permeable, all three compounds enhanced staining in *S. aurantiacum* cells, especially compound **4**, which increased the fluorescence intensity by more than 2-fold. In *R. oryzae*, whereas compound **2** did not alter cell permeability, compounds **3** and **4** increased the fluorescence intensity 3-fold (Figure 6B).

### 2.10. Influence of Exogenous Ergosterol on the Activity of Synthetic Compounds

Since the fungal membrane seems to be affected by the synthetic compounds, we evaluated whether they could interact with ergosterol, one of the most important lipids in fungal cells. To assess this, purified ergosterol was added to RPMI (Roswell Park Memorial Institute) medium to analyze whether it could interfere with the activity of the compounds.

**Figure 7:**
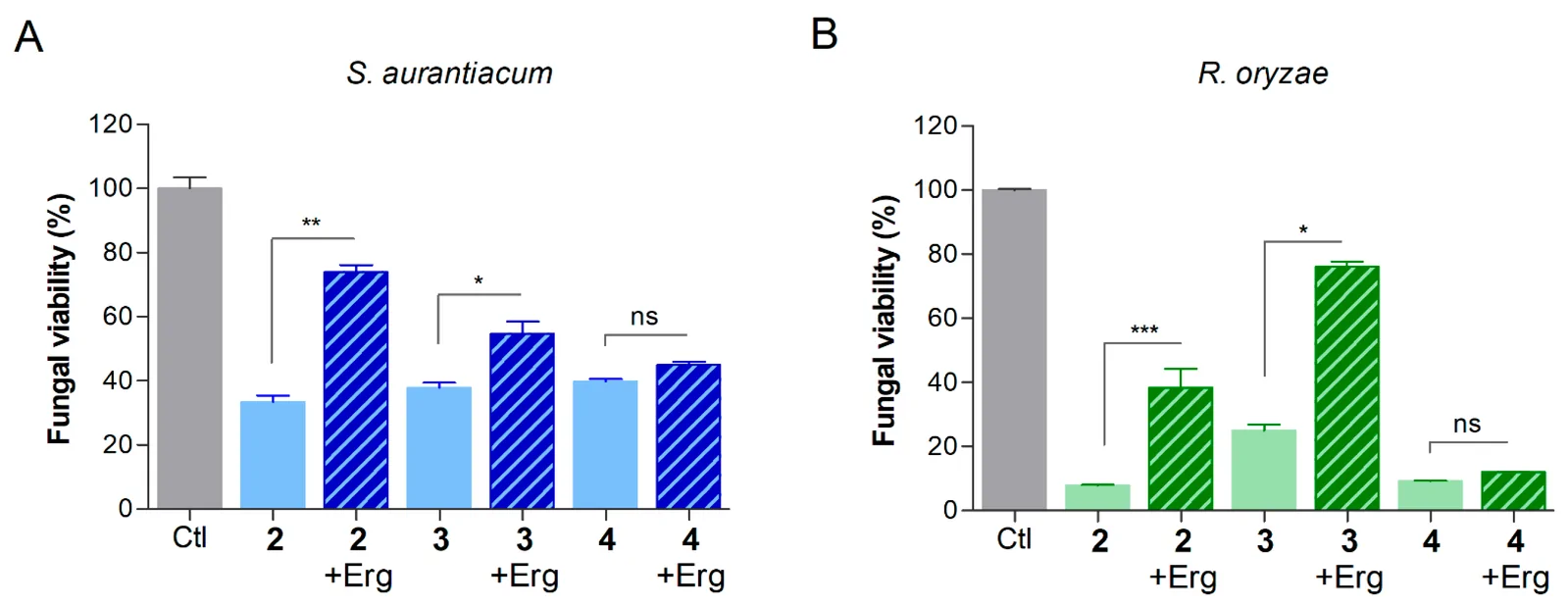
*S. aurantiacum* (**A**) and *R. oryzae* (**B**) susceptibility to compounds **2**, **3** and **4** in the absence or presence of exogenous ergosterol in culture media. Control (Ctl): positive control of fungal cells grown in the absence of any compounds. **2**: compound **2**; **3**: compound **3**; **4**: compound **4**. Erg: ergosterol. ns: not significant (*p* > 0.05), * *p* < 0.01, ** *p* < 0.001, *** *p* < 0.0001.

In both *S. aurantiacum* and *R. oryzae*, fungal viability increased after treatment with compounds **2** and **3**, but not with compound **4**, when exogenous ergosterol was present, suggesting that ergosterol may influence the antifungal activity of the compounds (Figure 7).

### 2.11. Alterations in Membrane Microdomains Caused by Synthetic Compounds

**Figure 8:**
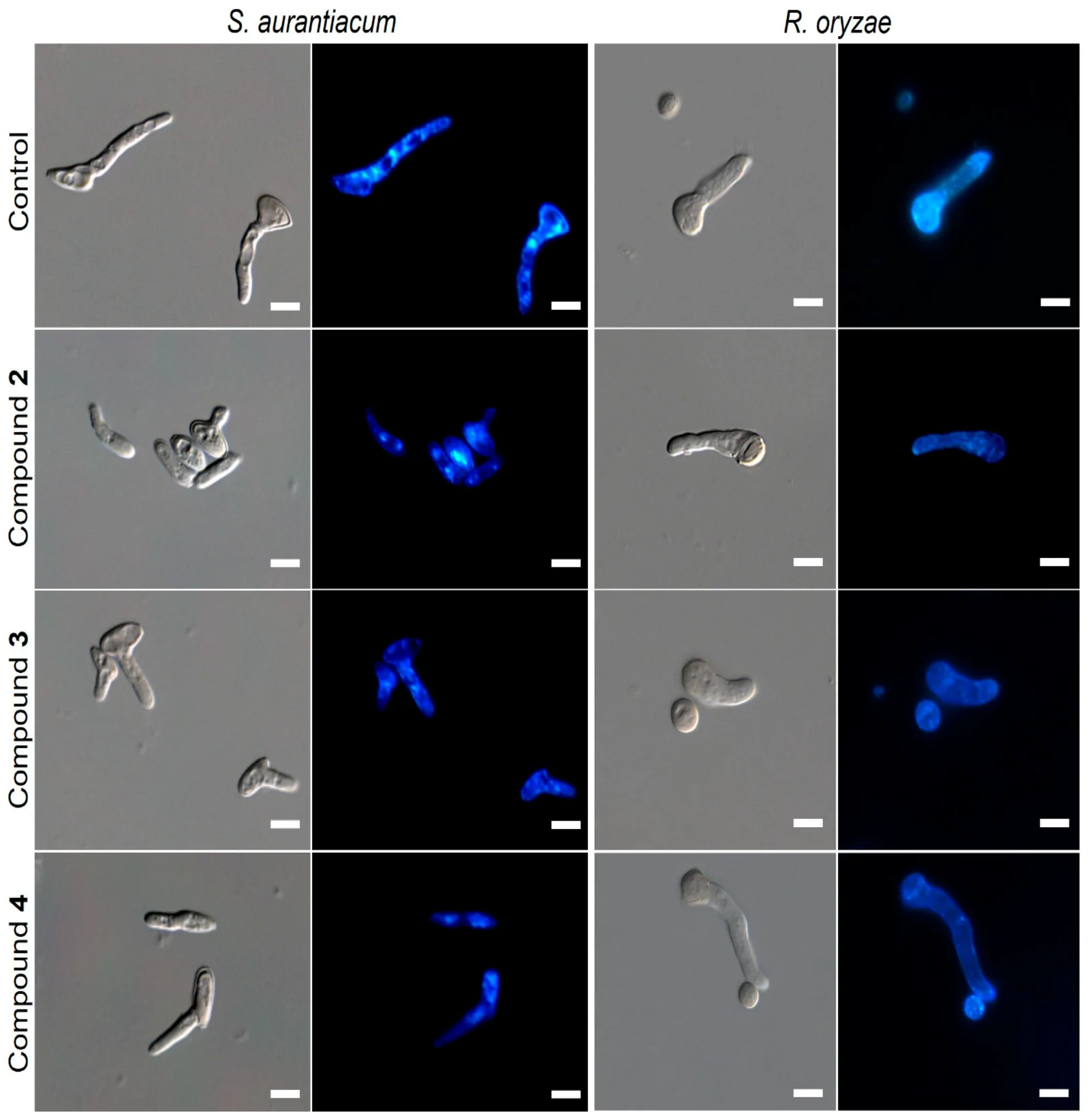
Fluorescence microscopy of *S. aurantiacum* and *R. oryzae* cells stained with filipin. Control represents cells grown in the absence of any compound. **2**: compound **2**; **3**: compound **3**; **4**: compound **4**. Scale bars: 10 μm.

Considering that the synthetic compounds may affect parameters related to the fungal membrane and cell surface, the distribution of sterol-enriched microdomains, also called lipid rafts, in the membranes of *S. aurantiacum* and *R. oryzae* was evaluated using filipin staining. Under control conditions, accumulation of lipid rafts was observed in regions where filipin staining was more intense (Figure 8). However, when cells were treated with all three compounds, the fluorescence caused by filipin was homogeneous, with no regions of accumulation along the cells, which suggests that the distribution of lipid rafts may be affected by the synthetic compounds (Figure 8).

### 2.12. Scanning Electron Microscopy (SEM) of Cells Treated with Synthetic Compounds

**Figure 9:**
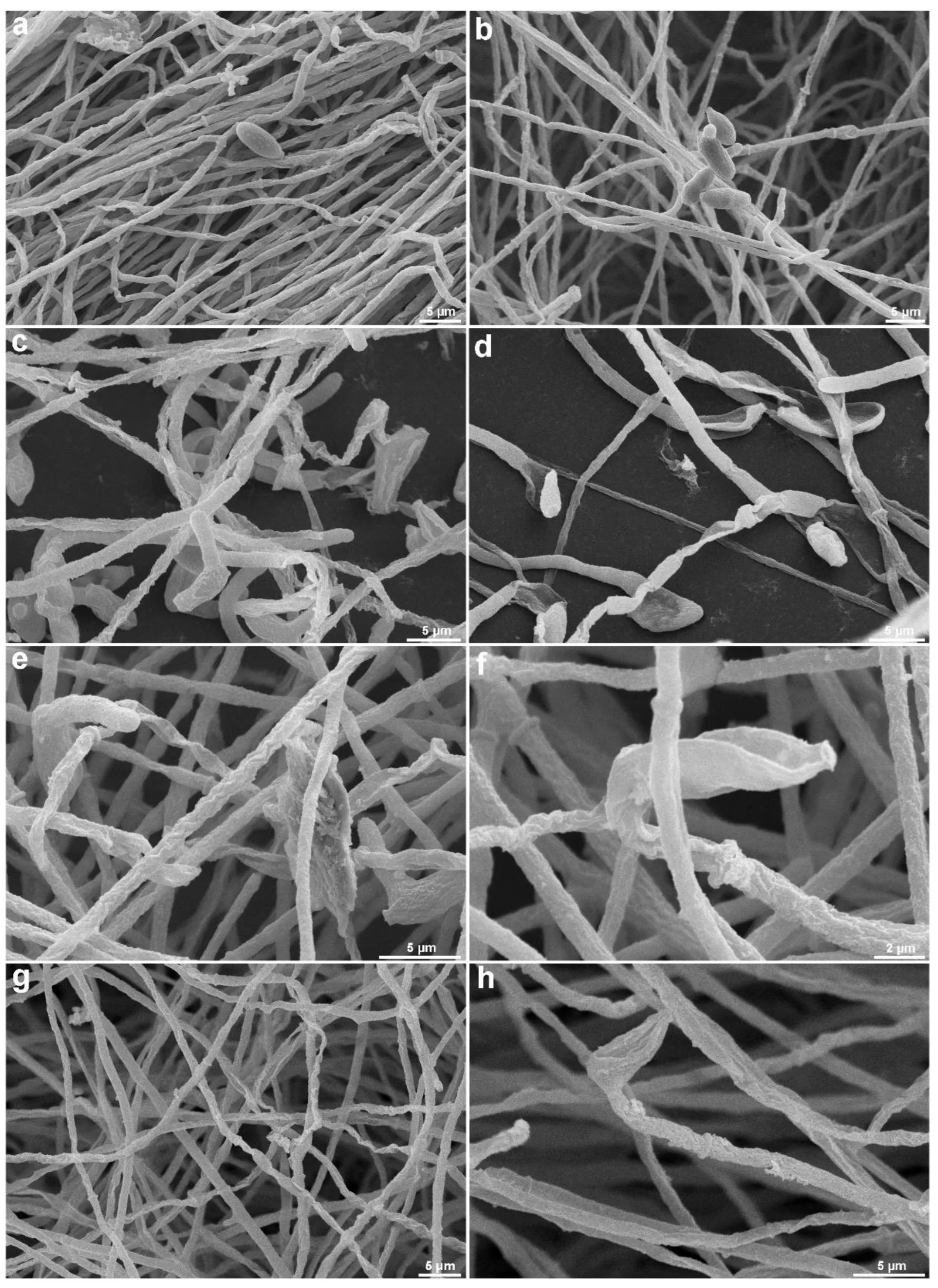
Scanning electron microscopy of *S. aurantiacum*. (**a**,**b**) Control in the absence of any compound. (**c**,**d**) Cells treated with compound **2**. (**e**,**f**) Cells treated with compound **3**. (**g**,**h**) Cells treated with compound **4**.

**Figure 10:**
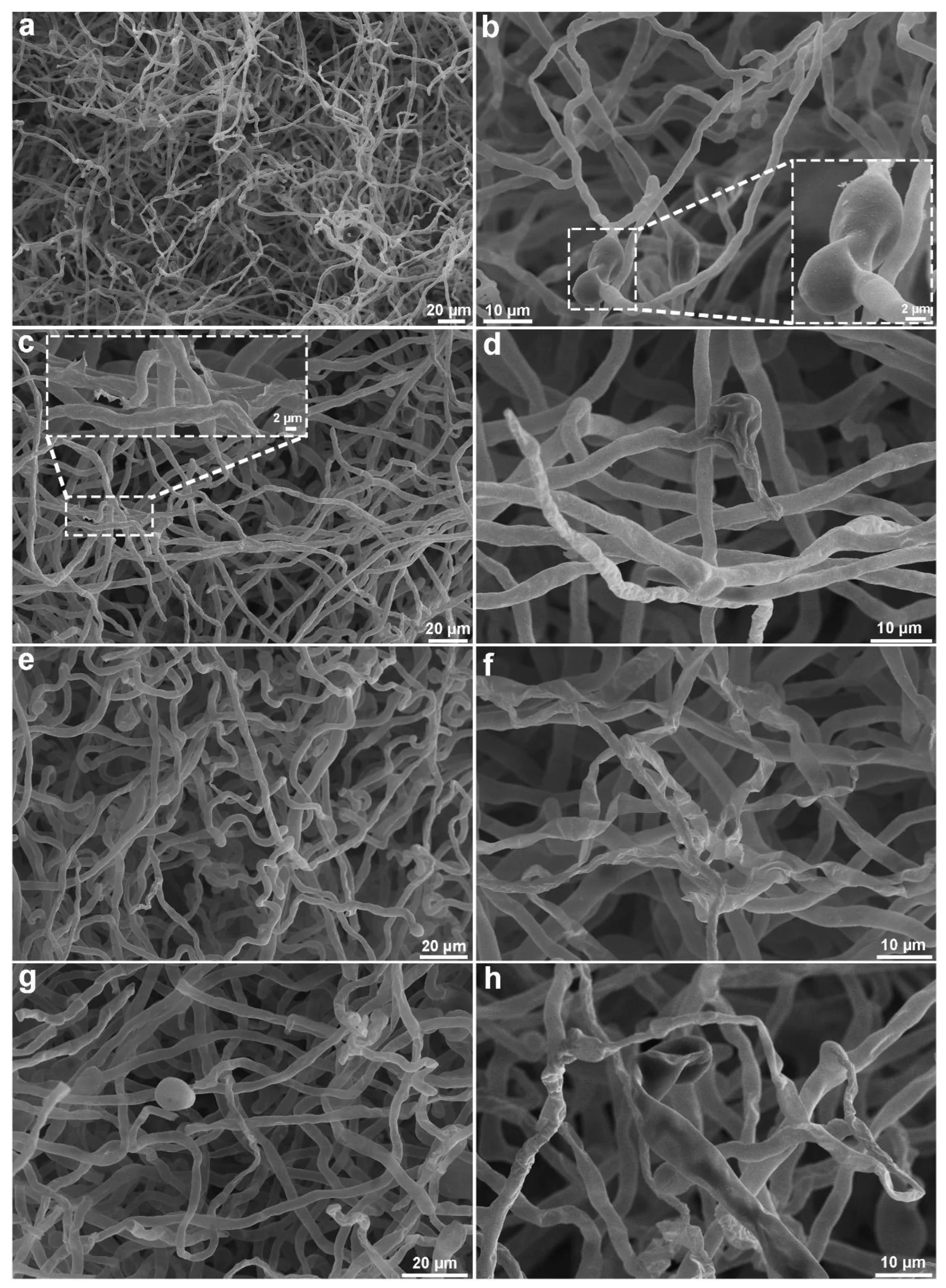
Scanning electron microscopy of *R oryzae*. (**a**,**b**) Control in the absence of any compound. (**c**,**d**) Cells treated with compound **2**. (**e**,**f**) Cells treated with compound **3**. (**g**,**h**) Cells treated with compound **4**.

To elucidate how these compounds affect fungal cells, SEM analysis was performed to evaluate fungal morphology. *S. aurantiacum* and *R. oryzae* were chosen as the representative species. *S. aurantiacum* grown in the absence of any compound showed thicker hyphae and the formation of elliptical conidia, typical of *Scedosporium* growth (Figure 9). When cells were treated with all three compounds, abnormal conidia (empty conidia) and some twisted hyphae were observed (Figure 9). In *R. oryzae*, all treatments resulted in twisted hyphae and abnormal mycelial morphology (Figure 10).

### 2.13. Transmission Electron Microscopy (TEM) of Cells Treated with Synthetic Compounds

**Figure 11:**
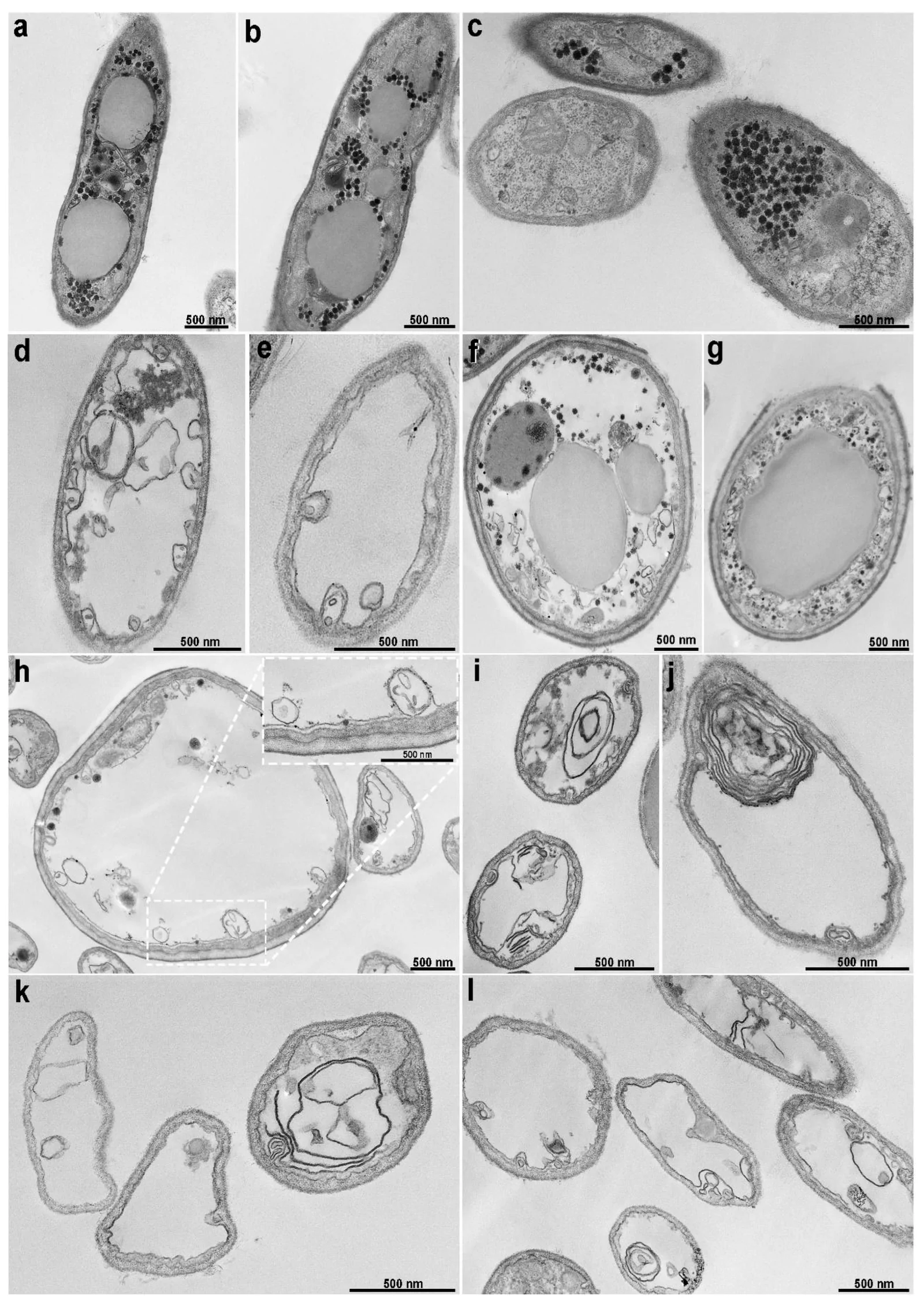
Transmission electron microscopy of *S. aurantiacum*. (**a**–**c**) Control in the absence of any compound. (**d**–**f**) Cells treated with compound **2**. (**g**,**h**) Cells treated with compound **3**. (**i**–**l**) Cells treated with compound **4**.

To investigate alterations in fungal cell wall and plasma membrane integrity, as well as in cytoplasmic content, a transmission electron microscopy analysis was performed. In *S. aurantiacum*, the control condition, in which cells were grown in the absence of any compound, displayed electron-dense intracytoplasmic material, vacuoles and a regular cell wall and plasma membrane (Figure 11a–c). When treated with compound **2**, cells presented an accumulation of vesicles on the inner face of the plasma membrane, and loss of cell wall integrity was observed (Figure 11d–f). After compound **3** treatment, cells displayed a disorganized cytoplasm, as well as an accumulation of vesicles on the inner face of the plasma membrane (Figure 11g,h). Regarding compound **4** treatment, a disorganized cytoplasm was also observed, as well as a less intense electron-dense cell surface (Figure 11i–l).

**Figure 12:**
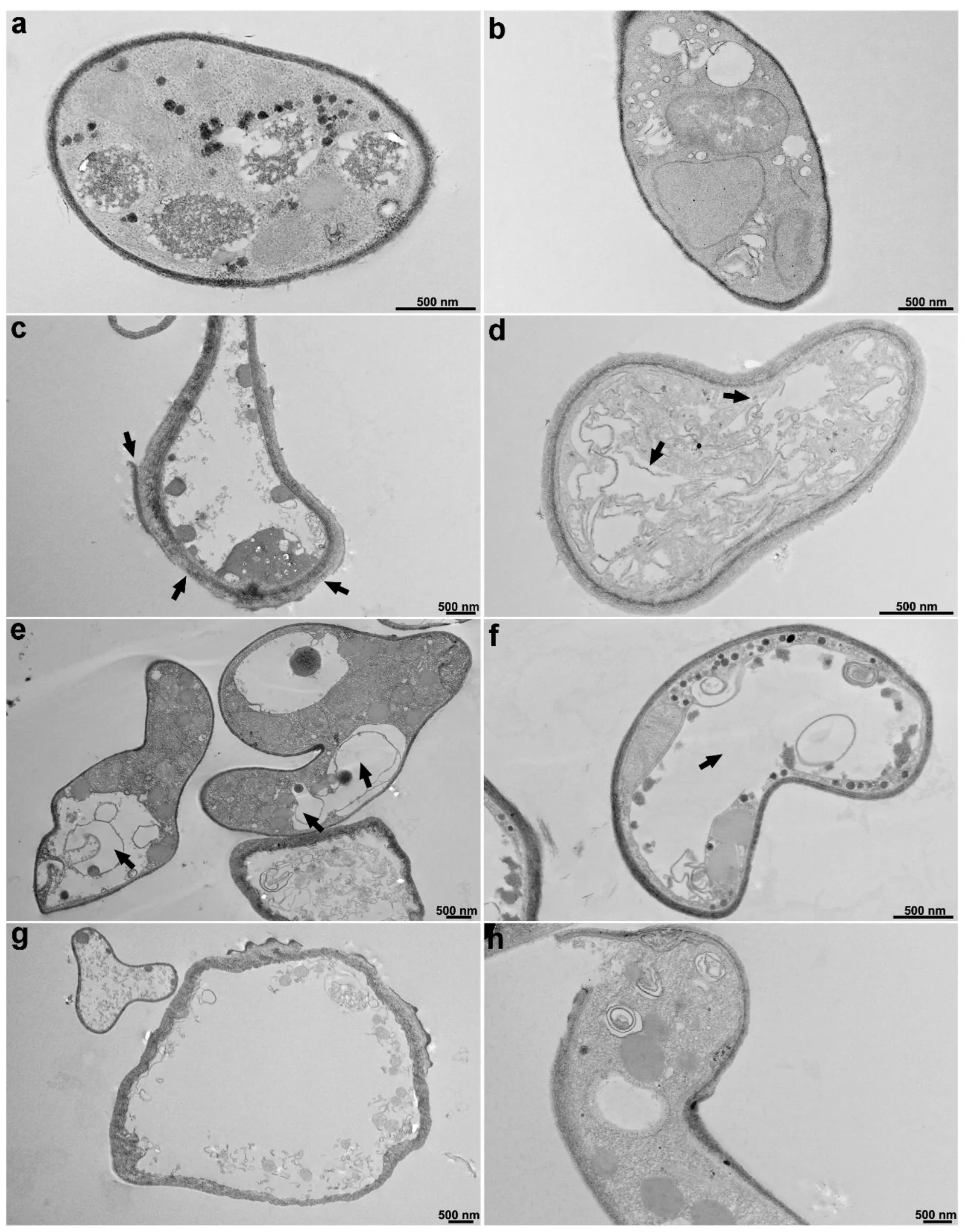
Transmission electron microscopy of *R. oryzae*. (**a**,**b**) Control in the absence of any compound. (**c**,**d**) Cells treated with compound **2**. (**e**,**f**) Cells treated with compound **3**. (**g**,**h**) Cells treated with compound **4**. In figures (**c**,**d**), arrows point damage cell surface and irregular vacuoles. In figures (**e**,**f**), arrows point to large vacuoles in the cytoplasm.

In *R. oryzae*, control cells displayed a regular cell surface, intracellular electron-dense material and an organized cytoplasm (Figure 12a,b). After treatment with compound **2**, loss of cell surface integrity and irregular vacuoles were observed (Figure 12c,d, black arrows). Compound **3** caused a large empty area in the cytoplasm (Figure 12e,f, black arrows), whereas treatment with compound **4** resulted in disruption of the cell surface (Figure 12g,h).

## 3. Discussion

The present study investigated the activity of a small series of synthetic organic compounds against two relevant groups of pathogenic filamentous fungi: the *Scedosporium*/*Lomentospora* complex and the order Mucorales. Among the compounds evaluated, three inhibited at least 60% of the growth, as well as biofilms, of *S. aurantiacum* and *R. oryzae*, species used as reference strains for the screening. These compounds, designated as **2**, **3**, and **4**, also exhibited activity against *S. boydii*, *L. prolificans*, and other *Rhizopus* species, demonstrating a spectrum of activity that includes some difficult-to-treat fungi, notably the virtually pan-resistant *L. prolificans* [13]. However, this activity did not extend to *M. velutinosus* and *Cunninghamella* sp., a result that reflects the notorious resistance of these fungi to conventional antifungals and repurposing candidates compared to other members of the order Mucorales [14,15,16].

Among the identified hits, compound **2** displayed the highest activity. Structurally, it consists of a 1,2-phenylene core symmetrically substituted with two *p*-fluorophenylselanyl groups. Although its synthesis was previously described [17], its antifungal activity against filamentous fungi had not been explored until now. This finding places compound **2** within the chemical space of organoselenium derivatives with antifungal activity, notably including ebselen and diphenyl diselenide [(PhSe)_2_] as the most studied examples [18]. Specifically, ebselen demonstrated activity against clinically relevant pathogens, such as *Rhizopus* spp. and *Scedosporium* spp. [19], while [(PhSe)_2_] exhibits activity against the latter [20]. Consequently, the activity reported here reinforces the pharmacological relevance of selenium-based scaffolds as a valuable platform for antifungal drug discovery.

Compounds **3** and **4** complete the list of hits, despite exhibiting higher MIC values than compound **2**. Compound **3** is an *N*-substituted benzamide incorporating a 3-(butyltellanyl)propyl side chain. In recent years, organotellurium derivatives have attracted considerable attention due to their broad biological properties, which include antimicrobial and antileishmanial activities [21,22,23], despite historical concerns regarding tellurium-associated toxicity [24]. Compound **4** also derives from the benzamide scaffold but differs in the nature of its *N*-substituted side chain: the 3-(butyltellanyl)propyl group present in compound **3** is replaced by a 3-hydroxy-4,4-dimethylpentyl chain. Despite these distinct side chains, compounds **3** and **4** displayed comparable MIC values against the panel of fungal species evaluated, suggesting that the benzamide scaffold represents a key antifungal pharmacophore.

Mechanistically, compounds **2**, **3**, and **4** were found to directly affect the fungal cell surface. The reduction in chitin content in *S. aurantiacum* and *R. oryzae*, as detected by decreased calcofluor white staining, suggests that these compounds alter cell wall composition. This effect likely compromises cell wall integrity, thereby disrupting osmotic protection, a hypothesis supported by the increased susceptibility of treated cells to NaCl. Ultrastructural analysis further revealed alterations in cell wall architecture and morphology in both species. Notably, this compromise of cell wall integrity distinguishes compound **2** from other organoselenium derivatives, such as LQA_78, which was reported to exert no impact on the cell wall of *Cryptococcus neoformans* [25]. Furthermore, the similar cell wall alterations promoted by compounds **3** and **4** indicate that this phenotype is independent of the tellurium incorporation in the *N*-alkyl side chain of the benzamide scaffold. In addition, these findings may explain the synergistic effect observed between the compounds and caspofungin against *S. aurantiacum*, as compromising cell wall integrity is known to enhance echinocandin activity, an effect also described for other cell wall-targeting agents such as nikkomycin Z and poacic acid [26,27].

Beyond the cell wall, these compounds also disrupted the fungal cell membrane, as evidenced by the reduction in Nile red staining and the increased susceptibility of treated *S. aurantiacum* and *R. oryzae* to SDS. Further evidence was provided by the extracellular DNA measurement and Sytox Green staining: in *S. aurantiacum*, all compounds induced membrane permeabilization, whereas in *R. oryzae* this effect was restricted to the *N*-substituted benzamides **3** and **4**, with compound **2** inducing no detectable permeabilization under the experimental conditions employed. This differential response may reflect species-specific differences in membrane lipid composition rather than an intrinsic property of compound **2**, given the distinctive lipid metabolic and compositional features reported for Mucorales, including lipid remodeling in response to stress [28,29,30,31,32,33]. Indeed, variations in membrane lipid composition can influence the activity of certain membrane-active compounds, as demonstrated for the aminoglycoside FG08, whose perturbation effects vary depending on the composition of model membranes [34]. Whether a similar mechanism explains the results for compound **2** in *R. oryzae* warrants further investigation.

Interestingly, part of the membrane disruption caused by compounds **2** and **3** appears to involve direct interaction with sterols, as the viability of treated cells increased in the presence of exogenous ergosterol, a pattern that resembles that of ergosterol-targeting agents [15,35]. Both compounds share the incorporation of heavy chalcogens, an observation that raises the possibility of selenium- or tellurium-mediated interactions with membrane sterols, although no studies to date have established this mechanism. A similar effect was not observed for compound **4**, suggesting that its membrane-disruptive activity does not depend on direct ergosterol binding. Regardless of these distinct interactions, cells treated with all three compounds exhibited reduced staining with filipin, a well-established sterol marker [36]. For compound **4**, this reduction may reflect a broader perturbation in membrane lipid organization, such as altered lipid packing, or a decrease in ergosterol content, possibly resulting from impaired sterol biosynthesis. For compounds **2** and **3**, this same effect is likely accompanied by direct ergosterol masking resulting from their interaction with the sterol, which potentially prevents filipin binding [36]. Of note, reduced filipin staining was also observed at the hyphal growing tips, regions typically enriched in lipid rafts. These sterol- and sphingolipid-rich microdomains are required for polarized growth [37,38,39], and the loss of localized fluorescence in treated cells suggests a compromise of these specialized membrane domains, potentially resulting in impaired fungal morphogenesis.

Collectively, the disruption of the cell membrane and lipid microdomains may contribute to the synergistic interactions observed between compounds **2**, **3**, or **4** and voriconazole or posaconazole. By perturbing membrane architecture, these compounds may facilitate the intracellular accumulation of azoles, thereby enhancing their activity, a mechanism similar to that described for the combination of colistin and the imidazopyrazoindole NPD827 with fluconazole [40,41]. For compound **3**, an additional mechanism may contribute to this effect: it acts as an efflux pump inhibitor, capable of reversing fluconazole resistance in *Saccharomyces cerevisiae* expressing Pdr5p and in *Candida albicans* expressing Cdr1p and Mdr1p [11,12]. Although the *S. aurantiacum* and *R. oryzae* isolates used in this study are susceptible to the azoles evaluated, basal efflux pump inhibition could further enhance azole activity, as supported by studies showing that the efflux pump inhibitor PAβN increased voriconazole activity against azole-susceptible isolates of *Aspergillus turcosus* and *Scedosporium* spp. [42,43]. Furthermore, our analysis showed that compounds **2** and **3** act synergistically with amphotericin B against *R. oryzae*, indicating that membrane-associated potentiation may also occur with non-azole antifungals.

Notably, the combinations of the compounds with current antifungal agents were classified as synergistic by the BLISS method but as additive or indifferent by the FICI method. This discrepancy reflects the distinct pharmacological models underlying the two approaches: BLISS is based on independence, whereas FICI is based on Loewe additivity, which assumes dose equivalence [44]. Consequently, the two approaches can yield different classifications for the same drug combination [45,46]. Thus, the synergistic interactions and their proposed mechanism require further validation.

Our findings also demonstrated that treatment of *S. aurantiacum* with all three compounds increased susceptibility to menadione, suggesting an impairment of the fungus’s ability to neutralize oxidative stress. For compounds **2** and **3**, their chalcogen-containing moieties may contribute to this effect, as some organoselenium and organotellurium compounds have been reported to perturb cellular redox homeostasis [47,48,49,50,51]. In contrast, neither of the compounds significantly altered menadione susceptibility in *R. oryzae*, suggesting that the contribution of oxidative stress to the antifungal effects of these compounds may vary among fungal species and requires further investigation.

Beyond antifungal activity, a favorable safety profile is an essential consideration in drug discovery, as toxicity remains a leading cause of attrition at all stages of development [52]. Our in silico analyses predicted that compounds **2**, **3**, and **4** possess a low risk of acute toxicity, as evidenced by LD_50_ values from GUSAR across multiple administration routes. Furthermore, compound **2** emerged as a particularly promising hit, showing no toxicity alerts in the Osiris Property Explorer. Although alerts for tumorigenicity in compound **3** and reproductive effects in compound **4** warrant attention, it is important to note that these computational predictions rely on structure-based fragment alerts and require in vivo validation. In addition, it is worth noting that fragment-based toxicity alerts for organochalcogenide compounds, particularly those containing tellurium, should be interpreted with caution, as training datasets may inadequately represent this chemical space. Therefore, the present work lacks in vitro and in vivo evaluations, and this limitation should be addressed in future studies to elucidate the toxicity of these compounds.

Ultimately, these compounds provide a structural basis for hit-to-lead optimization aimed at developing more potent antifungal agents against clinically relevant members of the *Scedosporium*/*Lomentospora* complex and *Rhizopus* spp. However, their effects on clinical isolates and their in vivo activity are key aspects to be investigated to demonstrate the potential of these compounds as antifungal agents.

## 4. Materials and Methods

### 4.1. Strains and Growth Conditions

The following strains were kindly provided by Sybren De Hoog, from the Westerdijk Fungal Biodiversity Institute, Utrecht, the Netherlands: *Scedosporium aurantiacum* CBS 136046 and *Scedosporium boydii* CBS 120157. *Lomentospora prolificans* FMR 3569 was kindly provided by Dr J. Guarro from the Unitat de Microbiologia, Facultat de Medicina e Institut d‘Estudis Avançats, Reus, Spain. *Rhizopus oryzae* UCP1295, *Rhizopus microsporus* var. *microsporus* UCP1304 and *Rhizopus stolonifer* UCP1300, isolated from the Brazilian Caatinga area, were supplied by Galba Maria de Campos-Takaki, from the Culture Collection (RENNORFUN) of the Catholic University of Pernambuco, Recife, Brazil. Clinical strains of *Mucor velutinosus* H136BO and *Cunninghamella* sp. B926 were supplied by Marcio Nucci from the Mycology Laboratory of the University Hospital, Universidade Federal do Rio de Janeiro.

For *Scedosporium* and *Lomentospora* growth, fungal stocks were kept in modified Sabouraud medium (0.5% yeast extract, 1% peptone, and 2% glucose monohydrate), whereas Potato Dextrose Broth (PDB; Neogen Culture Media, Lansing, MI, USA) was used for Mucorales culture. To obtain conidia for the experiments, cells were grown on modified Sabouraud agar plates or Potato Dextrose Agar (PDA; Neogen Culture Media, Lansing, MI, USA) for seven days at room temperature. The cell growth on the surface of the medium was scraped and suspended in sterile phosphate-buffered saline (PBS; 10 mM NaH_2_PO_4_, 10 mM Na_2_HPO_4_ and 150 mM NaCl, pH 7.2). The suspension was filtered, centrifuged and cells were counted in a Neubauer’s chamber before being used in the experiments.

### 4.2. Synthetic Compounds

The compounds screened for antifungal activity are synthetic organic structures, some containing tellurium, selenium or silicon (Figure 1). The synthesis procedures and spectroscopic characterization data are reported in the original publications from our group [17,53,54,55,56]. All compounds were stored in a desiccator at 4 °C, and stock solutions were prepared in dimethyl sulfoxide (DMSO; Tedia, Fairfield, OH, USA).

### 4.3. Antifungal Susceptibility Testing

Eight compounds were screened using *Scedosporium aurantiacum* CBS 136046 and *Rhizopus oryzae* UCP1295 as reference strains. Screening was performed at a concentration of 100 μg/mL of each compound diluted in RPMI 1640 medium (Sigma Aldrich Chemical Co., St. Louis, MO, USA) supplemented with 2% glucose and buffered with 3-(N-morpholino) propanesulfonic acid (MOPS) (0.165 mol/L, pH 7.2, hereafter referred to as ‘supplemented RPMI’) in 96-well microtiter plates. Voriconazole and posaconazole (Sigma-Aldrich, St. Louis, MO, USA), both at 5 μg/mL and supplemented RPMI with 0.5% DMSO were used as controls. Conidia (2 × 10^5^/mL) were added and incubated for 72 h at 37 °C in a 5% CO_2_ atmosphere. Fungal growth was quantified by optical density readings using a spectrophotometer (Bio-Rad, Hercules, CA, USA) at 600 nm. An inhibition of at least 50% was defined as a cut-off to select promising compounds with antifungal activity due to the resistance pattern of these fungi [57,58].

The fungal susceptibility to the active compounds found in the screening step was determined using the broth microdilution method, following the EUCAST protocols, with modifications [59]. Briefly, a serial dilution series (100–3.125 μg/mL) of each compound was prepared in 96-well microplates using supplemented RPMI 1640 medium. A conidia suspension (2 × 10^5^/mL) was added to each well and incubated for 72 h at 37 °C in a 5% CO_2_ atmosphere. Fungal growth was quantified by spectrophotometric readings (Bio-Rad, Hercules, CA, USA) at 600 nm, and the XTT (2,3-bis-(2-methoxy-4-nitro-5-sulfophenyl)-2H-tetrazolium-5-carboxanilide; Sigma-Aldrich, St. Louis, MO, USA) reduction assay was performed to assess cell viability [60]. The minimum inhibitory concentration (MIC) of each compound was defined as the lowest concentration that inhibited 50% of fungal growth.

### 4.4. Biofilm Assays

The effect of the selected compounds against fungal biofilms was analyzed according to Rollin-Pinheiro and colleagues [61]. *S. aurantiacum* CBS 136046 and *R. oryzae* UCP1295 were used as reference strains. Firstly, an adhesion step was performed by adding 200 μL from a standardized conidia suspension (1 × 10^7^/mL) to each well of a polystyrene microplate (NEST Biotechnology Co., Ltd., Wuxi, Jiangsu, China) and incubating for 1.5 h at 37 °C. Then, the supernatant containing non-adherent cells was removed, and supplemented RPMI 1640 medium was added, after which the adherent cells were incubated for 24 h at 37 °C to form biofilms. After 24 h of biofilm formation, the supernatant was removed and supplemented RPMI was added in the absence (positive control) or presence of the selected compounds (2 MIC and 1 MIC). An additional incubation of 24 h at 37 °C was performed to evaluate the anti-biofilm activity. Evaluation of fungal biofilms was carried out using the XTT-reduction assay.

### 4.5. Antifungal Drug Synergy Assay

Synergistic interactions were evaluated by the checkerboard method following the EUCAST guidelines [62]. *S. aurantiacum* CBS 136046 and *R. oryzae* UCP1295 were used as reference strains. Conidia (2 × 10^5^/mL) were grown in 96-well plates containing supplemented RPMI in the presence of selected compounds (100−3.125 μg/mL) combined with voriconazole (10−0.15 μg/mL) or caspofungin (40−0.625 μg/mL,) for *S. aurantiacum*, or posaconazole (20−0.3 μg/mL) or amphotericin B (40−0.625 μg/mL, Sigma-Aldrich, St. Louis, MO, USA) for *R. oryzae*. After incubation for 72 h at 37 °C, MIC was evaluated at 600 nm and cell viability was assessed by the XTT-reduction assay at 490 nm using a spectrophotometer (Bio-Rad, Hercules, CA, USA). An inhibition of at least 50% was defined as a cut-off for minimum inhibitory concentration (MIC).

Interactions were determined by the fractional inhibitory concentration index (FICI), which was calculated using the following formula: (MIC combined/MIC drug A alone) + (MIC combined/MIC drug B alone). The results were classified as: synergistic effect, FICI of <0.5; additive, FICI of >0.5–1.0; no effect, FICI of >1.0−4.0; antagonistic effect, FICI of >4.0 [63]. Bliss independence model was performed according to Meletiadis and colleagues and Zhao and colleagues [46,64]. The following formula was used to assess the drug interaction: *E*_exp_ = Ea + Eb − Ea × Eb, in which *E*_exp_ is the expected efficacy of the drug combination, Ea is the efficacy of drug A (synthetic compounds), and Eb is the efficacy of drug B (voriconazole, caspofungin, posaconazole or amphotericin B). The results were classified as: synergistic effect, *E*_obs_ > *E*_exp_; indifference, *E*_obs_ = *E*_exp_; antagonistic effect, *E*_obs_ < *E*_exp_.

### 4.6. Analysis of Fungal Cell Alterations

Alterations in fungal cells caused by the selected compounds were analyzed using the following fluorescent stains: Nile Red (Sigma-Aldrich, St. Louis, MO, USA) for the determination of neutral lipids, and calcofluor white (Sigma-Aldrich, St. Louis, MO, USA) to evaluate chitin content. Cells were grown in the absence (positive control) or presence of ½ MIC of the compounds for 48 h at 37 °C. Then, cells were stained for 1 h at 37 °C, protected from light, with 2 mg/mL of Nile Red or 25 μg/mL of calcofluor white [65,66]. Samples were washed three times to remove residual dye and suspended in PBS. Fluorescence intensity was measured using the SpectraMax M3 microplate reader (Molecular Devices, San Jose, CA, USA) under the following conditions: Nile Red at 550 nm (excitation) and 635 nm (emission); and calcofluor white at 350 nm (excitation) and 432 nm (emission).

The cell membrane permeability was determined by measuring the release of DNA into the culture supernatant [67]. Conidia (1 × 10^5^/mL) were grown in supplemented RPMI in the absence (positive control) or presence of 1 MIC of selected compounds for 48 h at 37 °C. Then, the cells were pelleted, and the supernatant was analyzed using a NanoDrop 2000 spectrophotometer (Thermo Fisher Scientific, Waltham, MA, USA) to quantify free DNA (260 nm). A sterile culture medium sample was used as a negative control.

Staining with Sytox Green (Invitrogen, Molecular Probes, Eugene, OR, USA) was also performed to evaluate cell permeability. Conidia (1 × 10^5^/mL) were grown in supplemented RPMI in the absence (positive control) or presence of 1 MIC of selected compounds. The cells were incubated for 48 h at 37 °C, and then washed three times. Next, the samples were stained with Sytox Green (5 μM) for 1 h at room temperature, protected from light. After washing three times, fluorescence intensity was measured using the SpectraMax M3 microplate reader (Molecular Devices, San Jose, CA, USA) at the emission/excitation wavelengths of 523 nm/504 nm [66].

To evaluate membrane microdomains, fungal cells were stained with filipin according to [38]. Briefly, conidia (1 × 10^5^/mL) were grown in 24-well plates containing supplemented RPMI in the absence or presence of 1× MIC of the selected compounds for 6 h at 37 °C. Then, the germinated conidia were washed three times with PBS and stained with 50 µg/mL of filipin (Sigma-Aldrich, St. Louis, MO, USA) for 2 h at room temperature, protected from light. After washing three times with PBS, the cells were observed using a fluorescence microscope (Axioplan Imager 2, Carl Zeiss, Oberkochen, Germany).

### 4.7. Susceptibility to Cell Stressors

To evaluate fungal susceptibility to cell stressors after treatment with sub-inhibitory concentrations of the selected compounds, sodium dodecyl sulfate (SDS; Bio-Rad, Hercules, CA, USA), NaCl (Neon Comercial, São Paulo, Brazil) and menadione (Sigma-Aldrich, St. Louis, MO, USA) were used as membrane, osmotic and oxidative stressors, respectively. Conidia (1 × 10^5^/mL) were grown in 96-well plates containing RPMI in the presence of selected compounds (½ MIC) for 24 h and then incubated for an additional 24 h with either sub-inhibitory concentrations of SDS (45 μg/mL), NaCl (1%) or menadione (30 μM). The positive control consisted of cells grown in the absence of compounds or stressors. After incubation at 37 °C in 5% CO_2_, cell viability was measured using the XTT-reduction assay, and readings were obtained using a spectrophotometer (Bio-Rad, Hercules, CA, USA) at 490 nm [15].

### 4.8. Susceptibility in the Presence of Exogenous Ergosterol

Fungal susceptibility to the selected compounds in the presence of exogenous ergosterol (Sigma-Aldrich, St. Louis, MO, USA) was determined following Xisto and colleagues [15]. Conidia (2 × 10^5^/mL) were incubated in the presence of the MIC of each compound. Whereas fungi incubated only with each compound were used as controls for the MIC values, cells were also grown with each compound using RPMI supplemented with purified ergosterol (100 μg/mL) to assess their susceptibility to the compounds in the presence of exogenous ergosterol. After 72 h of incubation at 37 °C in 5% CO_2_, cell viability was measured at 490 nm using a spectrophotometer (Bio-Rad, Hercules, CA, USA).

### 4.9. Scanning Electron Microscopy (SEM)

To analyze morphological alterations, fungal cells were cultured in the absence (positive control) or presence of ½ MIC of the compounds for 48 h at 37 °C. After treatment, the cells were fixed in 2.5% glutaraldehyde in 0.1 M sodium cacodylate buffer (pH 7.2) for 2 h at room temperature. Samples were then washed in the same buffer and post-fixed in 1% osmium tetroxide prepared at a 1:1 ratio in PBS for 30 min. After post-fixation, the samples were dehydrated in a graded ethanol series (15, 30, 50, 70, 90 and 100%; 30 min at each step), dried in a critical-point dryer (Bal-Tec AG, Pfäffikon, Switzerland) using CO_2_, sputter-coated with a 3–5 nm gold layer using a Denton metallizer (Denton Vacuum LLC, Moorestown, NJ, USA), and examined using a JEOL JSM-6701 scanning electron microscope (JEOL, Tokyo, Japan).

### 4.10. Transmission Electron Microscopy (TEM)

For ultrastructural analysis, fungal cells were cultured in the absence (positive control) or presence of ½ MIC of the compounds for 48 h at 37 °C. Then the samples were fixed as described for SEM and post-fixed in a solution containing 1% osmium tetroxide, 5 mM CaCl_2_ and 0.8% potassium ferrocyanide for 1 h at room temperature. Dehydration was performed in a graded acetone series (30 min at each step), followed by gradual infiltration and embedding in Spurr resin. Polymerization was carried out at 60 °C. Ultrathin sections (~60 nm) were obtained using an ultramicrotome (Leica Microsystems, Wetzlar, Germany) and collected on 300-mesh copper grids. Sections were contrasted with uranyl acetate and lead citrate, air-dried, and examined using a Hitachi HT7800 transmission electron microscope (Hitachi, Tokyo, Japan). All electron microscopy analyses were performed at the Rudolf Barth Electron Microscopy Platform, Instituto Oswaldo Cruz (IOC/Fiocruz), Rio de Janeiro, Brazil.

### 4.11. In Silico Toxicity Prediction Methodology

The software Osiris Property Explorer [68] was employed to provide qualitative predictions for mutagenicity, tumorigenicity, irritant, and reproductive effects, based on the detection of toxicophoric fragments. Each compound was submitted using its SMILES (Simplified Molecular Input Line Entry System) notation, and results were expressed as “Yes” or “No”, in the presence or absence of a toxicity risk, respectively. The following SMILES notations were used. Compound **2**: FC(C=C1)=CC=C1[Se]C2=CC=CC=C2[Se]C3=CC=C(F)C=C3; Compound **3**: CCCC[Te]CCCN([H])C(C1=CC=CC=C1)=O; Compound **4**: O=C(N([H])CCC(O)C(C)(C)C)C1=CC=CC=C1; Voriconazole: C[C@@H](C1=NC=NC=C1F)[C@@](O)(C2=CC=C(F)C=C2F)CN3N=CN=C; Posaconazole: O=C1N([C@@H](CC)[C@@H](O)C)N=CN1C2=CC=C(N3CCN(C4=CC=C(OC[C@@H]5CO[C@@](CN6N=CN=C6)(C7=CC=C(F)C=C7F)C5)C=C4)CC3)C=C2; Amphotericin B: O=C([C@H]1[C@]2([H])C[C@@H](O[C@@H]3O[C@H](C)[C@@H](O)[C@H](N)[C@@H]3O)/C=C/C=C/C=C/C=C/C=C/C=C/C=C/[C@H](C)[C@@H](O)[C@@H](C)[C@H](C)OC(C[C@H](O)C[C@H](O)CC[C@@H](O)[C@H](O)C[C@H](O)C[C@](O2)(O)C[C@@H]1O)=O)O.

Quantitative toxicity predictions were obtained using GUSAR (General Unrestricted Structure-Activity Relationships) [69]. This software was trained on a dataset of about 10,000 chemical structures and provides predictions of rat acute toxicity considering four routes of administration (intraperitoneal, intravenous, oral, and subcutaneous). Compounds were also submitted using SMILES notation, and results were expressed as LD_50_ (mg/kg). For both software programs, voriconazole, posaconazole, and amphotericin B were used as controls.

### 4.12. Statistical Analyses

All experiments were performed in triplicate, in three independent experimental sets. Statistical analyses were performed using GraphPad Prism version 5.00 for Windows (GraphPad Software, San Diego, CA, USA). One-way ANOVA was used for multiple comparisons using Tukey’s test (Tukey post-test), and a *t*-test was used for two-group comparisons. A 90% or 95% confidence interval was determined in all experiments.

## 5. Conclusions

This study identified three synthetic organic compounds with promising antifungal activity against clinically relevant Scedosporium/Lomentospora and Mucorales species, including the difficult-to-treat Lomentospora prolificans. Compounds **2**, **3**, and **4** impaired fungal growth, biofilm viability, cell wall integrity, membrane organization, and sterol-associated processes, indicating a multifactorial mode of action. Among them, compound **2** showed the most promising overall profile, combining the highest antifungal activity with favorable in silico toxicity predictions. These findings support compounds **2**–**4**, particularly compound **2**, as promising scaffolds for the development of new antifungal agents, but the limitations addressed in the Discussion need to be explored in the future. Further studies are warranted to elucidate their molecular targets, evaluate their efficacy against clinical isolates and other pathogenic fungi, investigate their in vivo activity and analyze their safety and selectivity in appropriate experimental models.

## Figures and Tables

**Table 1:** Screening of synthetic compounds against *S. aurantiacum* and *R. oryzae*. All compounds were tested at 100 μg/mL.

Compounds	% of Inhibition	
	*S. aurantiacum*	*R. oryzae*
**1**	14.6	46.8
**2**	74.8	60.3
**3**	73.0	66.3
**4**	73.3	70.1
**5**	35.6	52.2
**6**	30.7	63.0
**7**	46.4	56.5
**8**	27.9	58.9

**Table 2:** Minimum inhibitory (MIC) and fungicidal (MFC) concentrations of compounds 2, 3 and 4 against different *Scedosporium* and Mucorales species.

Fungi	Compounds μg/mL					
	2		3		4	
	MIC	MFC	MIC	MFC	MIC	MFC
*S. aurantiacum*	12.5	>100	50	>100	50	>100
*S. boydii*	25	>100	100	>100	50	>100
*L. prolificans*	12.5	>100	100	>100	100	>100
*R. oryzae*	12.5	>100	50	>100	50	>100
*R. stolonifer*	12.5	>100	100	>100	100	>100
*R. microsporus*	12.5	>100	100	>100	100	>100
*M. velutinosus*	>100	>100	>100	>100	>100	>100
*Cunninghamella* sp.	100	>100	100	>100	100	>100

**Table 3:** Interaction between compounds **2**, **3** or **4** with voriconazole, caspofungin, posaconazole or amphotericin B against *S. aurantiacum* and *R. oryzae* analyzed according to the FICI method.

*S. aurantiacum*				
Compounds	μg/mL			FICI
	MIC alone	MIC combined		
**2**	12.5	Vori/**2**	0.31/12.5	2.0 (I)
**3**	50	Vori/**3**	0.15/25	1.0 (Ad)
**4**	50	Vori/**4**	0.31/50	2.0 (I)
Voriconazole	0.31	Caspo/**2**	6.25/6.25	0.75 (Ad)
Caspofungin	25	Caspo/**3**	25/50	2.0 (I)
		Caspo/**4**	25/50	2.0 (I)
*R. oryzae*				
Compounds	μg/mL			FICI
	MIC alone	MIC combined		
**2**	12.5	Posa/**2**	5.0/12.5	2.0 (I)
**3**	50	Posa/**3**	5.0/50	2.0 (I)
**4**	50	Posa/**4**	5.0/50	2.0 (I)
Posaconazole	5.0	AmphoB/**2**	0.31/1.56	0.37 (S)
Amphotericin B	1.25	AmphoB/**3**	1.25/50	2.0 (I)
		AmphoB/**4**	1.25/50	2.0 (I)

Vori: voriconazole; Caspo: caspofungin; Posa: posaconazole; AmphoB: amphotericin B; **2**: compound **2**; **3**: compound **3**; **4**: compound **4**; (I): indifferent effect; (Ad): additive effect; (S): synergistic effect. FICI method was used to evaluate drug interactions.

**Table 4:** Interaction between compounds **2**, **3** or **4** with voriconazole, caspofungin, posaconazole or amphotericin B against *S. aurantiacum* and *R. oryzae* analyzed according to the Bliss independence method.

*S. aurantiacum*								
Compounds	Efficacy of drugs alone (% of inhibition)		Efficacy of combined drugs (% of inhibition)					
			Voriconazole			Caspofungin		
	MIC_50_	½ MIC_50_	*E*_obs_	*E*_exp_	Δ*E*, % (interaction)	*E*_obs_	*E*_exp_	Δ*E*, % (interaction)
**2**	73.9	22.5	43.1	41.5	1.6 (S)	88.0	12.8	75.2 (S)
**3**	77.3	27.9	51.3	46.7	4.6 (S)	12.4	11.7	0.7 (S)
**4**	82.9	3.4	30.5	26.3	4.2 (S)	21.8	10.8	11.0 (S)
Voriconazole	91.2	29.3	NP	NP	NP	NP	NP	NP
Caspofungin	93.2	4.4	NP	NP	NP	NP	NP	NP
*R. oryzae*								
Compounds	Efficacy of drugs alone (% of inhibition)		Efficacy of combined drugs (% of inhibition)					
			Posaconazole			Amphotericin B		
	MIC_50_	½ MIC_50_	*E*_obs_	*E*_exp_	Δ*E*, % (interaction)	*E*_obs_	*E*_exp_	Δ*E*, % (interaction)
**2**	86.1	2.8	24.0	3.9	20.1 (S)	65.5	28.0	37.5 (S)
**3**	82.2	5.4	19.0	7.3	11.7 (S)	42.0	40.0	2.0 (S)
**4**	75.0	0.5	16.0	1.2	14.8 (S)	29.2	59.9	−30.7 (A)
Posaconazole	79.7	1.2	NP	NP	NP	NP	NP	NP
Amphotericin B	72.6	31.6	NP	NP	NP	NP	NP	NP

**2**: compound **2**; **3**: compound **3**; **4**: compound **4**; *E*_obs_: efficacy observed; *E*_exp_: efficacy expected; Δ*E*: efficacy variation; (S): synergism; (A): antagonism. BLISS method was used to evaluate drug interactions.

**Table 5:** Toxicity risk prediction of the compounds by Osiris Property Explorer.

Compound	Mutagenic	Tumorigenic	Irritant	Reproductive Effect
**2**	No	No	No	No
**3**	No	Yes	No	No
**4**	No	No	No	Yes
Voriconazole	No	No	No	No
Posaconazole	Yes	Yes	No	No
Amphotericin B	No	No	No	No

**Table 6:** Rat acute toxicity prediction of the compounds by GUSAR (mg/kg).

Compound	Rat IP LD_50_	Rat IV LD_50_	Rat Oral LD_50_	Rat SC LD_50_
**2**	567.4	93.6	1722.0	646.1
**3**	422.0	94.8	1076.0	1035.0
**4**	825.0	204.1	2059.0	1750.0
Voriconazole	709.3	154.9	501.0	471.1
Posaconazole	807.6	42.2	835.5	370.0
Amphotericin B	937.1	25.8	1031.0	323.2

IP, intraperitoneal; IV, intravenous; SC, subcutaneous; LD_50_, lethal dose to 50% of a given population.

## Data Availability

The original contributions presented in this study are included in the article. Further inquiries can be directed to the corresponding authors.

## References

[1] Pagano L., Fernández O.M. (2025). Clinical aspects and recent advances in fungal diseases impacting human health. J. Antimicrob. Chemother..

[2] Denning D.W. (2024). Global incidence and mortality of severe fungal disease. Lancet Infect. Dis..

[3] Neoh C.F., Chen S.C., Lanternier F., Tio S.Y., Halliday C.L., Kidd S.E., Kong D.C.M., Meyer W., Hoenigl M., Slavin M.A. (2024). Scedosporiosis and lomentosporiosis: Modern perspectives on these difficult-to-treat rare mold infections. Clin. Microbiol. Rev..

[4] Petrikkos G., Skiada A., Lortholary O., Roilides E., Walsh T.J., Kontoyiannis D.P. (2012). Epidemiology and clinical manifestations of mucormycosis. Clin. Infect. Dis..

[5] Liang M., Xu J., Luo Y., Qu J. (2024). Epidemiology, pathogenesis, clinical characteristics, and treatment of mucormycosis: A review. Ann. Med..

[6] Lewis R.E., Kontoyiannis D.P. (2013). Epidemiology and treatment of mucormycosis. Future Microbiol..

[7] Sarvestani A.S., Pishdad G., Bolandparvaz S. (2013). Predisposing Factors for Mucormycosis in Patients with Diabetes Mellitus; An Experience of 21 Years in Southern Iran. Bull. Emerg. Trauma..

[8] Song Y., Verweij P.E., Buil J.B., de Hoog S., Liu J., Guo J., Liu W., Li R. (2025). Analysis of Susceptibility and Drug Resistance of Antifungal Agents in Aspergillosis and Mucormycosis Patients: A Systematic Review. Mycoses.

[9] Tahiri G., Lax C., Cánovas-Márquez J.T., Carrillo-Marín P., Sanchis M., Navarro E., Garre V., Nicolás F.E. (2023). Mucorales and Mucormycosis: Recent Insights and Future Prospects. J. Fungi.

[10] Hoenigl M., Salmanton-García J., Walsh T.J., Nucci M., Neoh C.F., Jenks J.D., Lackner M., Sprute R., Al-Hatmi A.M.S., Bassetti M. (2021). Global guideline for the diagnosis and management of rare mould infections: An initiative of the European Confederation of Medical Mycology in cooperation with the International Society for Human and Animal Mycology and the American Society for Microbiology. Lancet Infect. Dis..

[11] Reis de Sá L.F., Toledo F.T., de Sousa B.A., Gonçalves A.C., Tessis A.C., Wendler E.P., Comasseto J.V., Dos Santos A.A., Ferreira-Pereira A. (2014). Synthetic organotelluride compounds induce the reversal of Pdr5p mediated fluconazole resistance in *Saccharomyces cerevisiae*. BMC Microbiol..

[12] Reis de Sá L.F., Toledo F.T., Gonçalves A.C., Sousa B.A., Dos Santos A.A., Brasil P.F., Duarte da Silva V.A., Tessis A.C., Ramos J.A., Carvalho M.A. (2017). Synthetic Organotellurium Compounds Sensitize Drug-Resistant *Candida albicans* Clinical Isolates to Fluconazole. Antimicrob. Agents Chemother..

[13] Pellon A., Ramirez-Garcia A., Buldain I., Antoran A., Martin-Souto L., Rementeria A., Hernando F.L. (2018). Pathobiology of Lomentospora prolificans: Could this species serve as a model of primary antifungal resistance?. Int. J. Antimicrob. Agents.

[14] Badali H., Cañete-Gibas C., McCarthy D., Patterson H., Sanders C., David M.P., Mele J., Fan H., Wiederhold N.P. (2021). Epidemiology and Antifungal Susceptibilities of Mucoralean Fungi in Clinical Samples from the United States. J. Clin. Microbiol..

[15] Xisto M., Rollin-Pinheiro R., Rochetti V.P., Castro-Almeida Y., Borba-Santos L.P., Santos-Freitas G., Cypriano J., Abreu F., Rozental S., Barreto-Bergter E. (2023). Miltefosine: A Repurposing Drug against Mucorales Pathogens. J. Fungi.

[16] Xisto M., Rollin-Pinheiro R., da Rosa P.C.M., Souza Santos J., Irmão N., Abib J.A.A., Rochetti V.P., de Castro Almeida Y., Santos Freitas G., Cypriano J. (2026). Auranofin and iodoquinol as promising repurposing drugs against filamentous fungi: Antifungal activity and cellular alterations. Microbiol. Spectr..

[17] Toledo F.T., Marques H., Comasseto J.V., Raminelli C. (2007). The diorgano dichalcogenides addition to benzyne under mild conditions. Tetrahedron Lett..

[18] Friberg L.I.M., Blanchfield J.T., Blaskovich M.A.T. (2025). Organoselenium Compounds As a Promising Source of Novel Antimicrobial Agents. J. Med. Chem..

[19] Wall G., Chaturvedi A.K., Wormley F.L., Wiederhold N.P., Patterson H.P., Patterson T.F., Lopez-Ribot J.L. (2018). Screening a Repurposing Library for Inhibitors of Multidrug-Resistant *Candida auris* Identifies Ebselen as a Repositionable Candidate for Antifungal Drug Development. Antimicrob. Agents Chemother..

[20] Loreto E.S., Mario D.A., Santurio J.M., Alves S.H., Nogueira C.W., Zeni G. (2011). In vitro antifungal evaluation and structure-activity relationship of diphenyl diselenide and synthetic analogues. Mycoses.

[21] Angeli A., Etxebeste-Mitxeltorena M., Sanmartín C., Espuelas S., Moreno E., Azqueta A., Parkkila S., Carta F., Supuran C.T. (2020). Tellurides Bearing Sulfonamides as Novel Inhibitors of Leishmanial Carbonic Anhydrase with Potent Antileishmanial Activity. J. Med. Chem..

[22] Borges F., Zugman T., Bandeira P., Dalmolin M., Scariot D., Garcia F., Oliveira A., Nakamura C., Piovan L. (2021). Complementary Performance of Organoselenides and Organotellurides as Antimicrobials Agents. J. Braz. Chem. Soc..

[23] Dalal M., Antil N., Kumar B., Garg S. (2024). Unveiling the therapeutic potential of organotellurium(IV) Schiff base complexes through structural elucidation, antimalarial, antioxidant and antimicrobial studies. J. Mol. Struct..

[24] Sári D., Ferroudj A., Semsey D., El-Ramady H., Brevik E.C., Prokisch J. (2024). Tellurium and Nano-Tellurium: Medicine or Poison?. Nanomaterials.

[25] De Jesus D.F.F., De Freitas A.L.D., De Oliveira I.M., De Almeida L.C., Bastos R.W., Spadari C.C., Melo A.S.A., Santos D.A., Costa-Lotufo L.V., Reis F.C.G. (2023). Organoselenium Has a Potent Fungicidal Effect on *Cryptococcus neoformans* and Inhibits the Virulence Factors. Antimicrob. Agents Chemother..

[26] Ganesan L.T., Manavathu E.K., Cutright J.L., Alangaden G.J., Chandrasekar P.H. (2004). In-vitro activity of nikkomycin Z alone and in combination with polyenes, triazoles or echinocandins against Aspergillus fumigatus. Clin. Microbiol. Infect..

[27] Piotrowski J.S., Okada H., Lu F., Li S.C., Hinchman L., Ranjan A., Smith D.L., Higbee A.J., Ulbrich A., Coon J.J. (2015). Plant-derived antifungal agent poacic acid targets β-1,3-glucan. Proc. Natl. Acad. Sci. USA.

[28] Müller C., Neugebauer T., Zill P., Lass-Flörl C., Bracher F., Binder U. (2018). Sterol Composition of Clinically Relevant Mucorales and Changes Resulting from Posaconazole Treatment. Molecules.

[29] Ali B., Chauhan A., Kumar M., Kumar P., Carolus H., Lobo Romero C., Vergauwen R., Singh A., Banerjee A., Prakash A. (2024). A Comprehensive Analysis of the Lipidomic Signatures in Rhizopus delemar. J. Fungi.

[30] Dimitriew W., Espana J.D.A., Krüger T., Kniemeyer O., Panagiotou G., Schuster S., Brakhage A.A., Voigt K., Schäuble S. (2026). Genome-scale metabolic modelling of the priority fungal pathogen Lichtheimia corymbifera. Microlife.

[31] Pan X., Liu H., Liu J., Wang C., Wen J. (2016). Omics-based approaches reveal phospholipids remodeling of Rhizopus oryzae responding to furfural stress for fumaric acid-production from xylose. Bioresour. Technol..

[32] Danilova O.A., Ianutsevich E.A., Kochkina G.A., Tereshina V.M. (2025). Adaptation of the psychrophilic *Mucor psychrophilus* (Mucorales, Mucoromycota) to lower temperatures and under conditions of heat and osmotic shocks. Fungal Biol..

[33] Muszewska A., Okrasińska A., Steczkiewicz K., Drgas O., Orłowska M., Perlińska-Lenart U., Aleksandrzak-Piekarczyk T., Szatraj K., Zielenkiewicz U., Piłsyk S. (2021). Metabolic Potential, Ecology and Presence of Associated Bacteria Is Reflected in Genomic Diversity of Mucoromycotina. Front. Microbiol..

[34] Shrestha S., Grilley M., Fosso M.Y., Chang C.W., Takemoto J.Y. (2013). Membrane lipid-modulated mechanism of action and non-cytotoxicity of novel fungicide aminoglycoside FG08. PLoS ONE.

[35] Escalante A., Gattuso M., Pérez P., Zacchino S. (2008). Evidence for the mechanism of action of the antifungal phytolaccoside B isolated from *Phytolacca tetramera* Hauman. J. Nat. Prod..

[36] Van Leeuwen M.R., Smant W., de Boer W., Dijksterhuis J. (2008). Filipin is a reliable in situ marker of ergosterol in the plasma membrane of germinating conidia (spores) of Penicillium discolor and stains intensively at the site of germ tube formation. J. Microbiol. Methods.

[37] Alvarez F.J., Douglas L.M., Konopka J.B. (2007). Sterol-rich plasma membrane domains in fungi. Eukaryot. Cell.

[38] Rollin-Pinheiro R., Rochetti V.P., Xisto M., Liporagi-Lopes L.C., Bastos B., Rella A., Singh A., Rozental S., Del Poeta M., Barreto-Bergter E. (2019). Sphingolipid biosynthetic pathway is crucial for growth, biofilm formation and membrane integrity of *Scedosporium boydii*. Future Med. Chem..

[39] Rochetti V.P., Rollin-Pinheiro R., de Oliveira E.B., Xisto M., Barreto-Bergter E. (2020). Glucosylceramide Plays a Role in Fungal Germination, Lipid Raft Organization and Biofilm Adhesion of the Pathogenic Fungus *Scedosporium aurantiacum*. J. Fungi.

[40] Bibi M., Murphy S., Benhamou R.I., Rosenberg A., Ulman A., Bicanic T., Fridman M., Berman J. (2021). Combining Colistin and Fluconazole Synergistically Increases Fungal Membrane Permeability and Antifungal Cidality. ACS Infect. Dis..

[41] Revie N.M., Iyer K.R., Maxson M.E., Zhang J., Yan S., Fernandes C.M., Meyer K.J., Chen X., Skulska I., Fogal M. (2022). Targeting fungal membrane homeostasis with imidazopyrazoindoles impairs azole resistance and biofilm formation. Nat. Commun..

[42] Parent-Michaud M., Dufresne P.J., Fournier E., Folch B., Martineau C., Moreira S., Doucet N., De Repentigny L., Dufresne S.F. (2020). Prevalence and mechanisms of azole resistance in clinical isolates of *Aspergillus* section Fumigati species in a Canadian tertiary care centre, 2000 to 2013. J. Antimicrob. Chemother..

[43] Mello T.P., Ramos L.S., Andrade V.V., Torres-Santos E.C., Lackner M., Branquinha M.H., Santos A.L.S. (2025). Elucidating the augmented resistance profile of *Scedosporium*/*Lomentospora* species to azoles in a cystic fibrosis mimic environment. J. Antimicrob. Chemother..

[44] Lederer S., Dijkstra T.M.H., Heskes T. (2019). Additive Dose Response Models: Defining Synergy. Front. Pharmacol..

[45] Meletiadis J., Pournaras S., Roilides E., Walsh T.J. (2010). Defining fractional inhibitory concentration index cutoffs for additive interactions based on self-drug additive combinations, Monte Carlo simulation analysis, and in vitro-in vivo correlation data for antifungal drug combinations against *Aspergillus fumigatus*. Antimicrob. Agents Chemother..

[46] Meletiadis J., Andes D.R., Lockhart S.R., Ghannoum M.A., Knapp C.C., Ostrosky-Zeichner L., Pfaller M.A., Chaturvedi V., Walsh T.J. (2022). Multicenter Collaborative Study of the Interaction of Antifungal Combinations against *Candida* Spp. by Loewe Additivity and Bliss Independence-Based Response Surface Analysis. J. Fungi.

[47] Comparsi B., Meinerz D.F., Franco J.L., Posser T., Prestes Ade S., Stefanello S.T., dos Santos D.B., Wagner C., Farina M., Aschner M. (2012). Diphenyl ditelluride targets brain selenoproteins in vivo: Inhibition of cerebral thioredoxin reductase and glutathione peroxidase in mice after acute exposure. Mol. Cell Biochem..

[48] de Jesus F.E.A., de Carvalho Maia O.A., Dos Reis A.C., de Sousa Moura A., Mendes A.N., Dos Santos Marciel G.L., da Cruz Esteves J., Coêlho S.G.C., de Sá Guimarães Nolêto I.R., Barreto do Nascimento M.L.L. (2026). A mechanistic approach about oxidative, mutagenic, and cytotoxic effects of an organotellurane. Naunyn Schmiedebergs Arch. Pharmacol..

[49] He J., Li D., Xiong K., Ge Y., Jin H., Zhang G., Hong M., Tian Y., Yin J., Zeng H. (2012). Inhibition of thioredoxin reductase by a novel series of bis-1,2-benzisoselenazol-3(2H)-ones: Organoselenium compounds for cancer therapy. Bioorg. Med. Chem..

[50] da Costa L.S., de Jesus D.F.F., Dunkl Brujas L., de Oliveira I.M., Bastos R.W., Melo A.S.A., Santos D.A., Stefani H.A., Ishida K. (2026). An Organoselenium Compound Induces Death in *Cryptococcus gattii* Yeast by Apoptosis and Necrosis and Shows Antifungal Efficacy in Galleria mellonella. ACS Omega.

[51] Thangamani S., Eldesouky H.E., Mohammad H., Pascuzzi P.E., Avramova L., Hazbun T.R., Seleem M.N. (2017). Ebselen exerts antifungal activity by regulating glutathione (GSH) and reactive oxygen species (ROS) production in fungal cells. Biochim. Biophys. Acta Gen. Subj..

[52] Kramer J.A., Sagartz J.E., Morris D.L. (2007). The application of discovery toxicology and pathology towards the design of safer pharmaceutical lead candidates. Nat. Rev. Drug Discov..

[53] Keppler A.F., Gariani R.A., Lopes D.G., Comasseto J.V. (2009). Lithium butylchalcogenolate induced Michael-aldol tandem sequence: Easy and rapid access to highly functionalized organochalcogenides and unsaturated compounds. Tetrahedron Lett..

[54] Sousa B.A., Keppler A.F., Gariani R.A., Comasseto J.V., Dos Santos A.A. (2012). Metallic chalcogenolates mediated modular Michael-aldol cascade reaction: An easy route to multi-functionalized chalcogenides and Morita–Baylis–Hillman adducts. Tetrahedron.

[55] Sousa B.A., Dos Santos A.A. (2012). A Facile, Versatile, and Mild Morita–Baylis–Hillman-Type Reaction for the Modular One-Pot Synthesis of Highly Functionalized MBH Adducts. Eur. J. Org. Chem..

[56] Vargas F., Toledo F., Comasseto J. (2010). N-Functionalized organolithium compounds via tellurium/lithium exchange reaction. J. Braz. Chem. Soc..

[57] Rollin-Pinheiro R., Xisto M., de Castro-Almeida Y., Rochetti V.P., Borba-Santos L.P., Fontes Y.D.S., Ferreira-Pereira A., Rozental S., Barreto-Bergter E. (2023). Pandemic Response Box^®^ library as a source of antifungal drugs against *Scedosporium* and *Lomentospora* species. PLoS ONE.

[58] Xisto M., Rollin-Pinheiro R., de Castro-Almeida Y., Dos Santos-Freitas G.M.P., Rochetti V.P., Borba-Santos L.P., da Silva Fontes Y., Ferreira-Pereira A., Rozental S., Barreto-Bergter E. (2023). Promising Antifungal Molecules against Mucormycosis Agents Identified from Pandemic Response Box(^®^): In Vitro and In Silico Analyses. J. Fungi.

[59] Taj-Aldeen S.J., Salah H., Al-Hatmi A.M., Hamed M., Theelen B., van Diepeningen A.D., Boekhout T., Lass-Flörl C. (2016). In vitro resistance of clinical Fusarium species to amphotericin B and voriconazole using the EUCAST antifungal susceptibility method. Diagn. Microbiol. Infect. Dis..

[60] Mello T.P., Aor A.C., Gonçalves D.S., Seabra S.H., Branquinha M.H., Santos A.L. (2016). Assessment of biofilm formation by *Scedosporium apiospermum*, *S. aurantiacum*, *S. minutisporum* and *Lomentospora prolificans*. Biofouling.

[61] Rollin-Pinheiro R., de Meirelles J.V., Vila T.V.M., Fonseca B.B., Alves V., Frases S., Rozental S., Barreto-Bergter E. (2017). Biofilm Formation by *Pseudallescheria*/*Scedosporium* Species: A Comparative Study. Front. Microbiol..

[62] Subcommittee on Antifungal Susceptibility Testing (AFST) of the ESCMID European Committee for Antimicrobial Susceptibility Testing (EUCAST) (2008). EUCAST Technical Note on the method for the determination of broth dilution minimum inhibitory concentrations of antifungal agents for conidia-forming moulds. Clin. Microbiol. Infect..

[63] Brilhante R.S.N., Araújo G.D.S., Fonseca X., Guedes G.M.M., Aguiar L., Castelo-Branco D., Cordeiro R.A., Sidrim J.J.C., Pereira Neto W.A., Rocha M.F.G. (2020). Antifungal effect of anthraquinones against *Cryptococcus neoformans*: Detection of synergism with amphotericin B. Med. Mycol..

[64] Zhao W., Sachsenmeier K., Zhang L., Sult E., Hollingsworth R.E., Yang H. (2014). A New Bliss Independence Model to Analyze Drug Combination Data. J. Biomol. Screen..

[65] de Oliveira E.B., Xisto M., Rollin-Pinheiro R., Rochetti V.P., Barreto-Bergter E. (2020). Peptidorhamnomannans from *Scedosporium* and *Lomentospora* Species Display Microbicidal Activity Against Bacteria Commonly Present in Cystic Fibrosis Patients. Front. Cell Infect. Microbiol..

[66] Rollin-Pinheiro R., Almeida Y.C., Rochetti V.P., Xisto M., Borba-Santos L.P., Rozental S., Barreto-Bergter E. (2021). Miltefosine Against *Scedosporium* and *Lomentospora* Species: Antifungal Activity and Its Effects on Fungal Cells. Front. Cell Infect. Microbiol..

[67] Spadari C.C., Vila T., Rozental S., Ishida K. (2018). Miltefosine Has a Postantifungal Effect and Induces Apoptosis in Cryptococcus Yeasts. Antimicrob. Agents Chemother..

[68] Sander T., Freyss J., von Korff M., Reich J.R., Rufener C. (2009). OSIRIS, an entirely in-house developed drug discovery informatics system. J. Chem. Inf. Model..

[69] Lagunin A., Zakharov A., Filimonov D., Poroikov V. (2011). QSAR Modelling of Rat Acute Toxicity on the Basis of PASS Prediction. Mol. Inf..

